# Nerolidol: A Sesquiterpene Alcohol with Multi-Faceted Pharmacological and Biological Activities

**DOI:** 10.3390/molecules21050529

**Published:** 2016-04-28

**Authors:** Weng-Keong Chan, Loh Teng-Hern Tan, Kok-Gan Chan, Learn-Han Lee, Bey-Hing Goh

**Affiliations:** 1 School of Pharmacy, Monash University Malaysia, 47500 Bandar Sunway, Selangor Darul Ehsan, Malaysia; wkcha29@student.monash.edu (W.-K.C.); lttan13@student.monash.edu (L.T.-H.T.); 2 Biomedical Research Laboratory, Jeffrey Cheah School of Medicine and Health Sciences, Monash University Malaysia, 47500 Bandar Sunway, Selangor Darul Ehsan, Malaysia; 3 Division of Genetics and Molecular Biology, Institute of Biological Sciences, Faculty of Science, University of Malaya, 50603 Kuala Lumpur, Malaysia; kokgan@um.edu.my; 4 Center of Health Outcomes Research and Therapeutic Safety (Cohorts), School of Pharmaceutical Sciences, University of Phayao, 56000 Phayao, Thailand

**Keywords:** *cis*-nerolidol, *trans*-nerolidol, sesquiterpene, essential oil, pharmacological activities

## Abstract

Nerolidol (3,7,11-trimethyl-1,6,10-dodecatrien-3-ol) is a naturally occurring sesquiterpene alcohol that is present in various plants with a floral odor. It is synthesized as an intermediate in the production of (3*E*)-4,8-dimethy-1,3,7-nonatriene (DMNT), a herbivore-induced volatile that protects plants from herbivore damage. Chemically, nerolidol exists in two geometric isomers, a *trans* and a *cis* form. The usage of nerolidol is widespread across different industries. It has been widely used in cosmetics (e.g., shampoos and perfumes) and in non-cosmetic products (e.g., detergents and cleansers). In fact, U.S. Food and Drug Administration (FDA) has also permitted the use of nerolidol as a food flavoring agent. The fact that nerolidol is a common ingredient in many products has attracted researchers to explore more medicinal properties of nerolidol that may exert beneficial effect on human health. Therefore, the aim of this review is to compile and consolidate the data on the various pharmacological and biological activities displayed by nerolidol. Furthermore, this review also includes pharmacokinetic and toxicological studies of nerolidol. In summary, the various pharmacological and biological activities demonstrated in this review highlight the prospects of nerolidol as a promising chemical or drug candidate in the field of agriculture and medicine.

## 1. Introduction

Ever since ancient times, medicinal plants have been explored and used as herbal medicines to treat many diseases [1]. With the advancement of technology, research on herbal medicine has intensified on the efforts to identify the bioactive compounds in medicinal plants that are responsible for their pharmacological and biological activities. Essential oils (EOs) are volatile, natural and complex bioactive compounds which are characterized by a strong odour, and their biological effects are known to be associated to a series of complex interactions with cells, tissues and whole organisms [2]. Besides its well-known application in aromatherapy [3], the uses of EO have been extended into the food, agriculture and pharmaceutical industries [4,5,6]. Among the plants that are rich in EOs are *Baccharis dracunculifolia* DC, *Elettaria cardamomum* (L.) Maton, *Momordica charantia* L., *Piper aleyreanum* C. DC and *Piper claussenianum* (Miq.) C. DC [7,8,9,10,11]. 

Nerolidol (3,7,11-trimethyl-1,6,10-dodecatrien-3-ol), also known as peruviol, is a naturally occurring sesquiterpene alcohol present in the EO of various plants with a floral odour [12,13]. Nerolidol was found to exist as one of the bioactive compounds responsible for the biological activities demonstrated by the EOs of the aforementioned plants.

Statistics showed that the global usage of nerolidol *per annum* ranges from 10 to 100 metric tonnes [14]. For instance, nerolidol is frequently incorporated in cosmetics (e.g., shampoos and perfumes) and non-cosmetic products (e.g., detergents and cleansers) [13]. Besides, nerolidol is also widely used in the food industry as a flavor enhancer in many food products since its approval by U.S. Food and Drug Administration as a safe food flavoring agent.

Principally, this article aims to review the diverse range of pharmacological and biological activities of nerolidol which include antioxidant, anti-microbial, anti-biofilm, anti-parasitic, insecticidal, anti-ulcer, skin penetration enhancer, anti-tumor, anti-nociceptive and anti-inflammatory properties. The review also covers the chemical structure, physical properties, and the biosynthesis pathway of nerolidol as the intermediate involved in the mechanisms responsible for protection of plants against herbivores and plant pathogens. This article also highlights the pharmacokinetic and toxicological properties of nerolidol in both *in vitro* and *in vivo* experimental models.

## 2. Chemical Structure and Physical Properties

**Figure 1:**
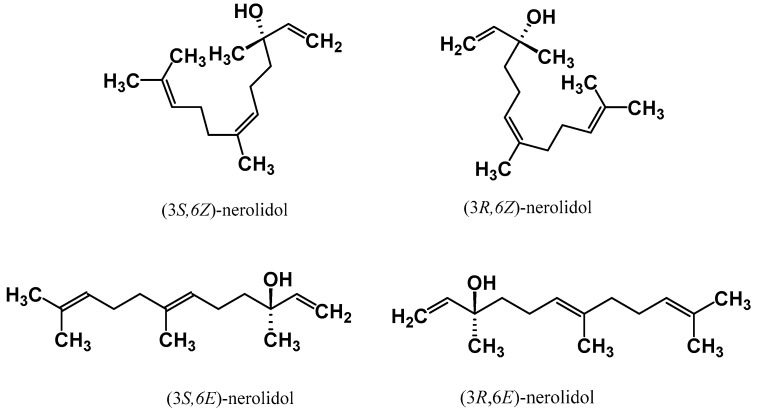
Chemical structures of the two enantiomers both for *cis*- and *trans*-isomers of nerolidol.

Nerolidol has four different isomeric forms which consist of two enantiomers and two geometric isomers [15]. The existence of these isomeric forms is due to the presence of a double bond at the C-6 position and the asymmetric center at the C-3 position. These isomeric forms of *cis*- and *trans*-nerolidol are illustrated in Figure 1. Besides, the synonyms for *cis*- and *trans*-nerolidol are listed in Table 1.

Like other sesquiterpene compounds, nerolidol has high hydrophobicity, thereby allowing easier penetration across the plasma membrane and interaction with intracellular proteins and/or intra-organelle sites [16]. The physical properties of nerolidol (isomer not specified) have been described by Lapczynski *et al.* [13] as follows:

(i) Physical description: A clear pale yellow to yellow liquid having a faint floral odor reminiscent of rose and apple.
(ii) Chemical formula: C_15_H_26_O
(iii) Flash point: >212° F; CC.
(iv) Boiling point: 276 °C.
(v) LogK_ow_ (calculated): 5.68.
(vi) Vapor pressure (calculated): 0.1 mm Hg 20 °C.
(vii) Specific gravity: 0.8744.
(viii) Water solubility (calculated): 1.532 mg/L at 25 °C.

## 3. Sources, Extraction and Analytical Methods of Nerolidol

Numerous extraction methods have been employed for extracting EOs from various plant samples [2]. The hydrodistillation method using the Clevenger-type apparatus appears as the most common method used for extracting nerolidol. Table 2 summarizes the different extraction methods and the yield of nerolidol from various parts of plants such as leaves, flowers, seeds, fruits, resins, twigs and woods. Based on the literature references, leaves are the most common source for extraction of nerolidol. In terms of the percentage of nerolidol in the leaf EO among different plant species, *Piper claussenianum* (Miq.) C. DC. has the highest percentage of *trans-*nerolidol (81.4%), followed by *Zanthoxylum hyemale* A.St.-Hil. (51.0%), *Zornia brasiliensis* Vogel (48.0%) and *Swinglea glutinosa* (Blanco) Merr. (28.4%) (Table 2).

Microclimatic and environmental factors such as species, season, location, climate, soil type, age of the leaves and the extraction method may influence the concentration of each constituents in EOs [17]. Seasonal variation is one of the main factors that influences the composition of EOs in plants [18,19,20] including the concentration of nerolidol. It was reported by Marques and Kaplan [19] that the harvested leaves from *Piper claussenianum* (Miq.) C. DC. yielded variable amounts of nerolidol during the year of 2009. The content of *trans-nerolidol* was higher during the Brazilian spring collection period (September, October and November, 87.0%, 94.0%, 92.0%, respectively) as compared to that during autumn collection period (March, April and May, 78.0%; 77.0%; 80.0%, respectively). Another study conducted by de Sousa *et al.* [20] has shown that the mean concentration of *trans*-nerolidol in the leaves of *Baccharis dracunculifolia* DC. was five fold higher in March 2005 (136.53 mg/100 g of plant) than that in July 2004 (25.03 mg/100 g of plant). All these findings provide important information to identify and determine the most appropriate harvest period to obtain the highest yield of nerolidol from different plants.

Gas chromatography-mass spectrometry (GC-MS) is the analytical method that is most commonly used to detect nerolidol [21]. This is because the boiling points of sesquiterpenes range from ~250 to 280 °C in which suitable for the gas-phase separation technique employed by GC-MS analysis [22,23]. Apart from GC-MS, liquid chromatography-mass spectrometry (LC-MS) method is also widely used due to its high sensitivity and high accuracy [24]. Recently, He *et al.* [25] suggested that LC-MS could be used for *in vivo* pharmacokinetic analysis of nerolidol due to its convenience and stability features. The study demonstrated that the lower limit lower quantification (LLOQ) of nerolidol using LC-MS was reported as 10 ng/mL [25]. On the other hand, another study reported the LLOQ of nerolidol as 3.5 ng/mL by using GC-MS [22]. These results suggest that GC-MS may be a more preferable detection method as it was shown to have higher sensitivity than LC-MS in detecting nerolidol.

In order to differentiatie the *cis*- and *trans*-isomers of nerolidol, the retention time of different LC-MS and GC-MS chromatography columns as well as the major peaks of the mass spectra (*m*/*z*) are the parameters used (Table 3). According to Table 3, *cis*-nerolidol displayed shorter retention times than *trans*-nerolidol regardless of the type of GC or LC column used. Besides retention time, one can also discriminate *cis*- from *trans*-nerolidol by referring to the retention indices (RIs) and RIs can be used for comparison across different chromatographic systems [53]. RIs, also known as Kováts retention indices, are frequently used along with mass spectrometry because the combination provides a more accurate identification of isomers, which is often difficult to be achieved by mass spectrometry alone [54]. The retention indices of different chromatographic columns of GC are shown in Table 3.

## 4. Industrial Synthesis of Nerolidol

**Scheme 1:**
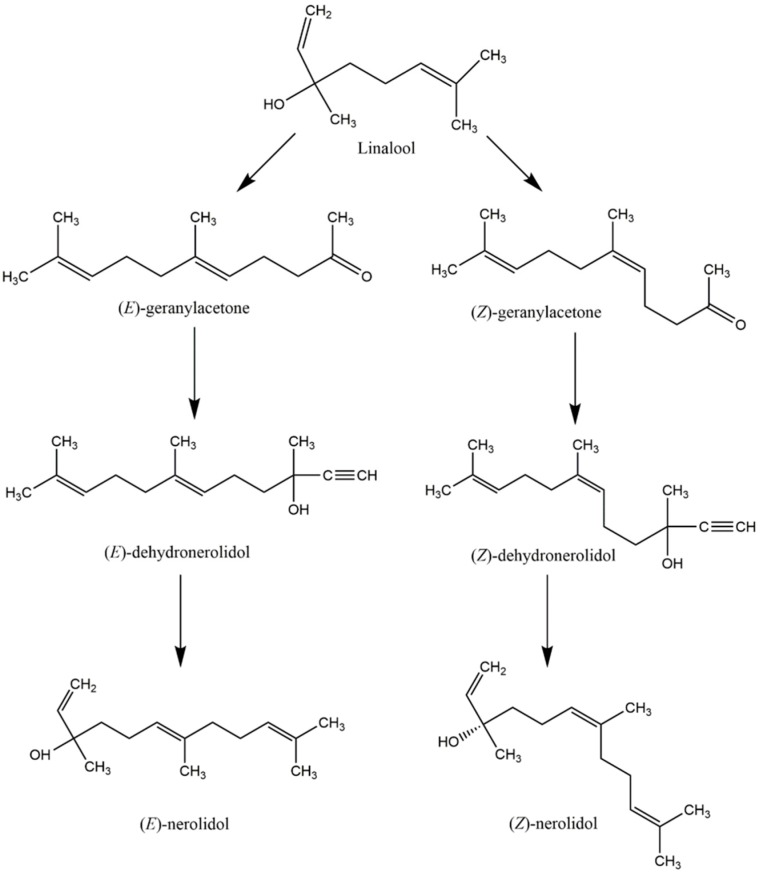
The overall chemical synthesis pathway of nerolidol to fulfill the demand of nerolidol in the industrial sector.

Chemical synthesis of nerolidol is required to increase its production in order to meet the growing industrial demand for nerolidol. Initially, nerolidol was synthesized as an intermediate in the chemical synthesis of geranyl esters from linalool [59]. The process began with the treatment of linalool with diketene or ethyl acetoacetate by the Carroll reaction to yield a mixture of (*E*)- and (*Z*)-geranylacetone [59]. Addition of acetylene to both (*E*)- and (*Z*)-geranylacetone led to the production of (*E*)- and (*Z*)-dehydronerolidol, respectively, which were selectively hydrogenated to *trans*- and *cis*-nerolidol, respectively, using a Lindlar catalyst [60]. The overall chemical synthesis of (*E*)- and (*Z*)-nerolidol is illustrated as shown in Scheme 1 as described by Nigmatov *et al.* [59].

Although nerolidol can be obtained via the aforementioned chemical reaction or isolation from natural sources, both methods suffer from the disadvantages that they are expensive and produce low yields of end products. In order to overcome these limitations, researchers have utilized eukaryotes such as yeast to produce higher yields of nerolidol. Therefore, a new method of nerolidol production has been developed and patented [61]. The method involved the cultivation of a yeast strain (particularly *Saccharomyces cerevisiae*, as it is a natural producer of farnesyl diphosphate (FDP)) lacking functional squalene synthase by modifying one *ERG9* squalene synthase gene. This was because the absence of functional squalene synthase prevented the conversion of FDP to squalene, therefore causing FDP to accumulate. The next step involved modifying the yeast to overexpress 3-hydroxy-3-methylglutaryl-coenzyme A (HMG CoA) reductase using an inducible promoter such as *GAL1* HMG CoA reductase, leading to a higher throughput of FDP. The last step involved growing the yeast in a synthetic medium which is lacking of uracil so that FDP can be fully hydrolyzed into nerolidol. Further shifting towards nerolidol production can be also enhanced by adjusting the pH of the medium to be more acidic either at the start, during or at the end of the growth cycle.

## 5. The Ecological Role and Biosynthesis of Nerolidol

Plant secondary metabolites (PSMs) are organic compounds that do not interfere with the primary metabolism of plants. Given that they mediate many ecological functions, PSMs are mainly secreted as plant defenses against herbivore and pathogen damages [62]. PSMs are stored either constitutively in inactive forms or induced in response to insect or microbe attack. To thwart off pathogens and herbivores, PSMs employ different chemical defensive strategies involving secondary metabolite pathways [62]. The first strategy consists of an indirect defense mechanism in which the plants confront herbivores indirectly by secreting herbivore-induced plant volatiles (HIPVs) to attract parasitoids and natural enemies of herbivores [63]. On the other hand, the direct defense mechanism employs another strategy, that is, toxic, volatile and non-volatile metabolites which are stored in specialized cells to be released or activated when plants are attacked by pathogens [64].

Among PSMs, terpenoids are the most structurally diverse group. For instance, monoterpenes and sesquiterpenes are the major volatile terpenoids released from plants [65]. Their function are diverse ranging from basic plant functions such as photosynthesis, respiration, growth and development, to playing role in plant defense mechanism to protect plants against herbivore and pathogen attacks [66].

In general, terpenoids are formed from the universal C5 precursor isopentenyl diphosphate (IPP) and its allylic isomer dimethylallyl diphosphate (DMAPP) [67]. Subsequently, condensation of IPP and DMAPP by prenyltransferases leads to the production of linear isoprenyl diphosphate precursors of many chain lengths such as geranyl diphosphate (GDP), FDP and geranylgeranyl diphosphate (GGDP). The allylic prenyldiphosphates of GDP, FDP and GGDP are then converted by terpene synthases (TPSs) to form monoterpenes (C_10_), sesquiterpenes (C_15_) and diterpenes (C_20_), respectively [68].

**Scheme 2:**
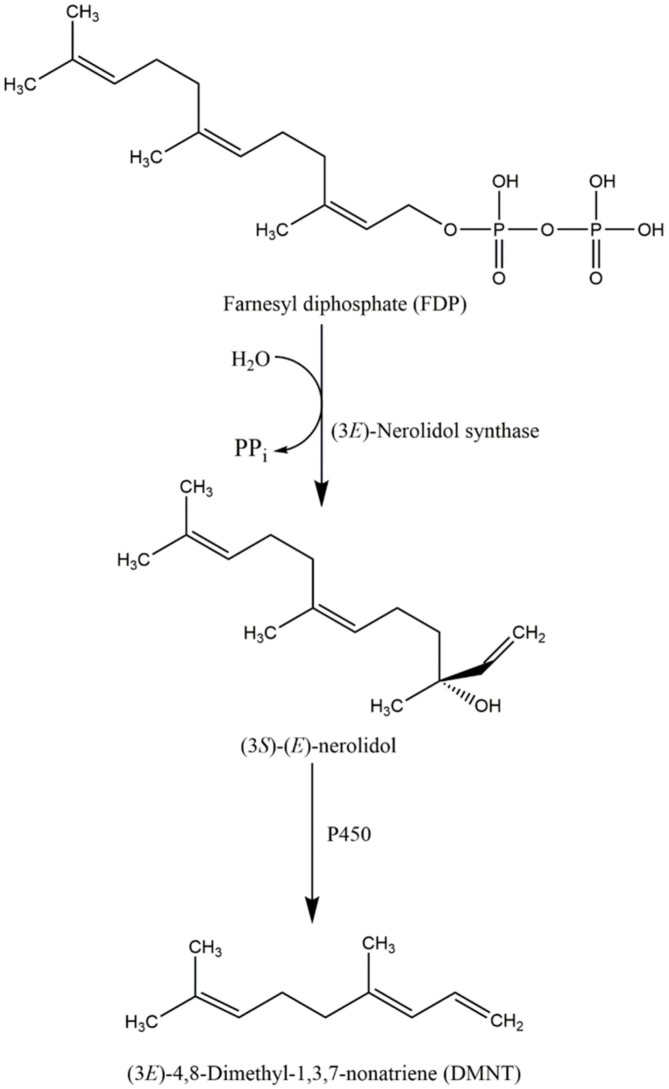
The biosynthesis pathway of (3*S*)-(*E*)-nerolidol as an intermediate product for the production of DMNT as an herbivore-induced volatile to protect the plant against herbivore damage.

With regard to the production of nerolidol, (*E*)-nerolidol synthase was recently found to be responsible for the conversion of FDP, the universal precursor of sesquiterpenes to (3*S*)-(*E*)-nerolidol. In snapdragon (*Antirrhinum majus* L.), Nagegowda *et al.* have recently purified two nerolidol/linalool synthases (AmNES/LIS-1/-2) that are responsible for the production of nerolidol and linalool. AmNES/LIS-1 is found in the cytosol and is responsible for nerolidol biosynthesis, whereas AmNES/LIS-2 is located in the plastids and is responsible for the formation of linalool [69]. Similar to snapdragon, (3*S*)-(*E*)-nerolidol synthase activities were demonstrated in maize [70]. Schnee *et al.* isolated the terpene synthase 1 (TPS1) enzyme, which is encoded by the maize *TPS1* gene to produce both 3*R*- and 3*S*-enantiomer of (*E*)-nerolidol [71]. Due to the stimulation of herbivore damage, the expression of *tps1* was increased by almost 8-fold, followed by the conversion of (*E*)-nerolidol to (3*E*)-4,8-dimethyl-1,3,7-nonatriene (DMNT). Taken all together, the conversion of (*E*)-nerolidol to DMNT is crucial due to the fact that DMNT acts as an herbivore-induced volatile to protect the plant against herbivore damage. The overall biosynthesis mechanism of nerolidol is illustrated in Scheme 2 as described by Bouwmeester *et al.* [72].

## 6. Pharmacological and Biological Activities of Nerolidol

**Figure 2:**
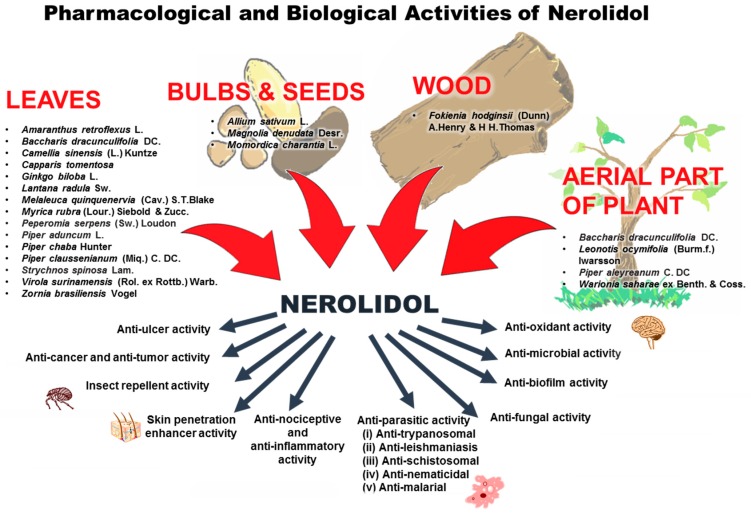
The source of extraction of nerolidol and an overview of the biological activities of nerolidol.

With the knowledge that nerolidol plays a very active role in the defense system of some plants, researchers have been interested to further explore various aspects of its pharmacological and biological activities. To date, various pharmacological and biological activities of nerolidol have been reported such as anti-microbial, anti-biofilm, anti-oxidant, anti-parasitic, skin-penetration enhancer, skin-repellent, anti-nociceptive, anti-inflammatory and anti-cancer. Table 4 summarizes the important information on the pharmacological and biological activities of nerolidol in different *in vitro* and *in vivo* models. Besides the pharmacological and biological activities of nerolidol, the sources of nerolidol extraction from various parts of plants are illustrated as well (Figure 2).

### 6.1. Antioxidant Activity

Reactive oxygen species (ROS) are formed by the incomplete reduction of oxygen during aerobic metabolism [110]. Superoxide anion (O_2_^–^), hydrogen peroxide (H_2_O_2_), and hydroxyl radicals (OH•) are some examples of ROS. Under normal circumstance or low level of oxidative stress, an in-built antioxidant defense system in the body helps the cells to counteract with any potential damages by detoxifying ROS with appropriate enzymes such as glutathione (GSH) reductase, GSH peroxidase, superoxide dismutase (SOD) and catalase. However, an imbalance in the antioxidant defense system or overproduction of free radicals which exceeds the detoxification capacity of cell may contribute to the onset of oxidative stress [111]. During oxidative stress, an elevation of intracellular levels of ROS was found to cause damage on biomolecules (lipids, proteins and DNA) [112]. Thus, high level of ROS is detrimental to cells. It is mainly due to the formation and accumulation of cellular damage resulted from oxidative stress and subsequently leading to the loss of cellular functions. If left untreated, it will result in many complications such as cancer, cardiovascular diseases and neurodegenerative disorders [113].

Given the fact that chemical compounds belonging to the sesquiterpene group are well known for their antioxidant properties [113,114], perhaps antioxidant activity may be expected from nerolidol. Furthermore, EOs containing nerolidol derived from medicinal plants were found to exhibit antioxidant activity, suggesting its plausible utilization as an antioxidant agent. Indeed, it has been demonstrated that nerolidol exhibits potent antioxidant properties in counterbalancing the effect of ROS by protecting the cells against oxidative damage to lipids, proteins and DNA [73,74,75]. According to a study conducted by Vinholes *et al.*, the antioxidant activity of *cis*-nerolidol was evaluated using 1,1-diphenyl-2-picrylhydrazine (DPPH) radical scavenging assay. The study revealed that *cis*-nerolidol exhibited DPPH scavenging activity [73]. In another study, *cis*-nerolidol was found to possess higher scavenging activity towards hydroxyl radicals with IC_50_ measured at 1.48 mM [73]. Due to the ability in scavenging several types of free radicals, *cis*-nerolidol was evidenced to protect Caco-2 cells against oxidative stress induced by tert-butyl hydroperoxide (tert-BuOOH), suggesting that nerolidol is a good antioxidant that exerts protection against oxidative damage [73]. Another study conducted by Vinholes *et al.* reported that *cis-*nerolidol mediated its strong antioxidant activity in protecting the hepatocytes through the inhibition of lipid peroxidation induced by tert-BuOOH, thereby 1mM of *cis*-nerolidol resulted in 36.50% ± 4.47% of malonaldehyde (MDA) reduction [74]. 

Besides the *in vitro* evidences displaying the antioxidant properties of nerolidol, an *in vivo* study by Nogueira Neto *et al.* demonstrated the neuroprotective effects of nerolidol (a mixture of *cis*- and *trans*-nerolidol) in adult male *Swiss* albino mice hippocampus against neuronal damages induced by oxidative stress [75]. The study demonstrated that significant decrease in MDA and nitrite levels were observed for the nerolidol group at doses of 25, 50 and 75 mg/kg when compared to saline, the negative control. Beside that, nerolidol also increased the antioxidant enzymatic activities of superoxide dismutase and catalase at doses of 25, 50 and 75 mg/kg. These observations suggest that nerolidol mediates a potent antioxidant activity by scavenging free radicals, preventing lipid peroxidation and enhancing the production of antioxidant enzymes in cells for protection against oxidative stress [115,116].

### 6.2. Antibacterial Activity

The decrease in the effectiveness of many antibiotics due to the rise of antimicrobial resistance is a global concern faced by the pharmaceutical, medical and food industries. Consequently, the number of infections caused by multidrug-resistant bacteria is increasing globally, leading to increased risk of mortality and morbidity [117]. Due to this, a large proportion of investments by pharmaceutical industries are being put into drug discovery research of new inhibitory compounds of microbiological, plant, or animal origin to be developed into potentially new anti-microbial drugs.

The studies showed that nerolidol exhibited potent antimicrobial activity against *Staphylococcus aureus* FDA 209P, 14 strains of methicillin-susceptible *S. aureus* (MSSA) and 20 strains of methicillin-resistant *S. aureus* (MRSA) with MIC values ranging from 512 to over 1024 μg/mL [76]. Besides, nerolidol possessed antibacterial activity against various strains of *Staphylococcus aureus* including MRSA by disrupting the cell membranes as indicated by the increased leakage of K^+^ ions from the bacterial cells [76,77,78]. The observed effects could be due to the presence of the long aliphatic chain in chemical structure of nerolidol. The hypothesis may comply with the findings of Togashi *et al.* [118] that the terpene alcohols with carbon chains of C10 to C12 (the numbering is started from the carbon atom connected to a hydroxyl group) was found to exhibit a strong antibacterial activity against *S. aureus* FDA209P. Since the carbon chain length of nerolidol is C12, it was shown to cause damage to the cell membrane, leading to the leakage of macromolecules and eventually cell lysis [78]. Besides causing membrane disruption, nerolidol was also found to interfere with genes which regulate the pathogenicity of the pathogens. For example, a study conducted by Lee *et al.* reported that *cis-*nerolidol present in the black pepper oil was responsible for the down-regulation of the α-hemolysin gene *hla* expression in *S. aureus* via quantitative real-time PCR analyses [45]*.*

In a similar way, EOs of *Momordica charantia* L. seed was found to exhibit strong antibacterial activity against *S. aureus* ATCC 6538 with MIC value of 125 μg/mL. The high content of *trans*-nerolidol was suggested to be responsible for the antibacterial activity demonstrated by the EOs of *Momordica charantia* L. seed [7]. Likewise, the study conducted on the antimicrobial activity of green tea flavor components by Kubo [79] has also shown that nerolidol as one of the ten major green tea flavor compounds, exhibited anti-microbial activity against *S. aureus* with MIC values of 200 μg/mL. Besides, nerolidol was also found to exert the strongest antibacterial activity against *Streptococcus mutans* when compared among the ten green tea flavor compounds with a MIC value of 25 μg/mL [79]. Meanwhile, nerolidol derived from the leaf EO of *Ginkgo biloba* (L.) demonstrated the highest antibacterial activity against *Salmonella enterica*, and *S. aureus* when compared to other EOs (isophytol, linalool, β-sitosterol acetate, β-sitosterol, stigmasterol, ergosterol, β-sitosterol-3-*O*-β-d-gluco-pyranoside and *Ginkgo biloba* polyprenols (GBP)) [31]. The evidences presented above may suggest nerolidol can be a good antibacterial agent particularly against *S. aureus*.

Besides the direct antibacterial action of nerolidol, nerolidol (*cis*-nerolidol and the racemic mixture of cis- and *trans*-isomers (1:1)) was found to potentiate the action of antibiotics, namely amoxicilline/clavulanic acid against *S. aureus* and amoxicilline/clavulanic acid, ceftadizine and imipenem against *Escherichia coli* [80]. The sensitization effect was also observed when nerolidol enhanced the susceptibility of *S. aureus* to ciprofloxacin, clindamycin, erythromycin, gentamicin, tetracycline, and vancomycin. Other than *S. aureus,* nerolidol was also found to enhance the susceptibility of *E. coli* ATCC 25922 to polymyxin B [81]. These findings were further supported by the experiment conducted by Simões *et al.* as it revealed that the treatment of nerolidol (racemic mixture of the *cis*- and *trans*-nerolidol) (1:1) potentiated the susceptibility of *E. coli* and *S. aureus* towards the antibiotics (ciprofloxacin, erythromycin, gentamicin and vancomycin), thereby resulted in significantly lower MIC concentrations [82]. Moreover, the study also demonstrated a moderate correlation between cell killing and permeabilization effects of nerolidol against both *S. aureus* and *E. coli*, suggesting that nerolidol exerted its action by modifying the bacterial outer layer as evidenced by the increased propidium iodide uptake [82]. All these observations suggest that nerolidol provides an alternative therapeutic option for the development of drug combinations that may be more effective in controlling multi-drug resistant bacteria. 

### 6.3. Anti-Biofilm Activity

Many bacteria are known to possess the ability to produce biofilm, which is defined as a community of microorganisms held together by a self-produced extracellular matrix and attached to living or inert surfaces such as polystyrene, glass, stainless steel and blood components in different environments [119]. Due to the complexity of the biofilm structure formation, microbial biofilms represent a significant challenge to the medical and pharmaceutical industries. The formation of biofilm induces microbial resistance to anti-microbial agents as well as to the body’s immune system. It has also been associated with the increased antibiotic resistance, thereby leading to biofilm-associated infections which complicate the treatment procedure. Therefore, there is a new trend in recent studies focusing on the evaluation of essential oils as potential inhibitors of biofilm formation. In the meantime, nerolidol was found to exhibit anti-biofilm activity against a number of pathogens. For example, a study conducted by Lee *et al.* has revealed that the EO of *Cananga odorata* (Lam.) Hook.f. & Thomson exhibited strong biofilm activity in a dose-dependent manner against the biofilm formation of *S. aureus* ATCC 6538 [45]. The anti-biofilm activity was attributed to the presence of *cis*- and *trans*-nerolidol in the essential oil of *C. odorata*. It was demonstrated that the *cis*-nerolidol at 0.01% (*v*/*v*) inhibited *S. aureus* biofilm formation by more than 80%, whereas *trans*-nerolidol at similar concentration exerted 45% inhibition. Another study conducted by Curvelo *et al.* revealed that the EO of *Piper claussenianum* (Miq.) C. DC., Piperaceae leaf (*trans*-nerolidol identified as the main component (81.4%) in this EO) decreases the formation of biofilm by *Candida albicans* for 30% and 50% after 24 and 48 incubation hours, respectively [32]. The same study also compared the anti-biofilm activities of the *cis,trans-*nerolidol and *cis-*nerolidol on the pre-formed biofilm by *C. albicans*. The study indicated that *cis,trans-*nerolidol resulted a stronger reduction in the viability of the mature biofilm than that of *cis*-nerolidol. Thus, it was suggested that *trans*-nerolidol, which was the main constituent in EO of Piper claussenianum (Miq.) C. DC., Piperaceae leaf, may responsible for the observed anti-biofilm activity in reducing the viability of the pre-formed biofilms.

### 6.4. Anti-Fungal Activity

Various anti-fungal agents have been developed to control the spread of fungal diseases such as candidiasis [120]. However, there are serious questions concerning the safety of these drugs due to their well-known side-effects as well as the possible development of antifungal drug resistance [121]. Therefore, the attention has been shifted to bio-prospecting the natural products to overcome or control fungal infections. In fact, EOs have been extensively studied and have been proven to be effective against fungal infections [122]. 

There are many evidences that support the effectiveness of nerolidol in exhibiting anti-fungal activity. *Trans*-nerolidol, which is a major component of leaf EO of *Piper claussenianum* (Miq.) C. DC., Piperaceae (81.4%), has been shown to exhibit fungicidal activity against *Candida albicans* with MIC values measured ranging from 0.24%–1.26% [32]. Similarly, the leaf EO also exerted a strong activity in the inhibition of germ-tube transformation of *Candida albicans* by 81% [32]. In another study, nerolidol has also been found to possess strong antifungal activity by distorting the hyphal growth of *Trichophyton mentagrophytes* at the concentration of 0.4 mg/mL [16]. Also, the growth of *T. mentagrophytes* was inhibited by nerolidol derived from green tea flavor with MIC value measured at 12.5 μg/mL [79].

Lee *et al.* reported a strong anti-fungal effect of nerolidol against *Microsporum gypseum* that causes dermatophytosis, a superficial infection in keratinized tissues including hair, nail and stratum corneum of skin [83]. Although nerolidol (0.5%–2%) was found to exhibit lower anti-fungal activity as compared to eugenol (0.01%–0.03%), the study showed that nerolidol was more effective in reducing the skin lesion than eugenol in guinea pig model. Moreover, histopathologic analysis revealed that animals treated with nerolidol had a lower degree of hyperkeratosis and inflammatory cell infiltration than non-treated animals.

Besides its anti-fungal effect against human pathogens, nerolidol also shows promising outcomes in controlling fungal infections in plants caused by phytopathogenic fungi. *Trans*-nerolidol, extracted from EO of *Lantana radula* Sw., has demonstrated to exhibit stronger fungistatic activity against the the phytopathogenic fungi *Corynespora cassiicola* than the EO extracted from *Lantana camara* [33]. Similarly, the leaf EO of *Piper chaba* Hunter which contained the *trans*-nerolidol as one of the major constituents, exhibited antifungal activity against phytopathogenic fungi such as *Fusarium oxysporum*, *Phytophthora capsici*, *Colletotrichum capsici*, *Fusarium solani* and *Rhizoctonia solani* with 55.1 to 70.3% growth inhibition and a MIC ranging from 125 and 500 µg/mL [34].

Znini *et al.* have also reported strong anti-fungal activity of *trans*-nerolidol extracted from EOs of aerial parts of *Warionia saharae* ex Benth. & Coss. against the three apple phytopathogenic fungi, *Alternaria* sp., *Penicillium expansum* and *Rhizopus stolonifer* causing the deterioration of apple by significantly inhibiting the mycelial growth of all strains tested. It was also found to inhibit the fungal spore production of *Alternaria* sp., *P. expansum* and *R. stolonifera* at the dosage of 1, 2 and 2 µL/mL air, respectively [26]. Besides this result, another study conducted by Pontin *et al.* has revealed strong antifungal activity of nerolidol in inhibiting mycelial growth and sclerotial production by ~85% and ~ 84%, respectively [84]. Nerolidol was also found to cause alterations in hyphal morphology and membrane permeability as demonstrated by hyphal shrinkage and partial distortion [84]. In addition, the study revealed an increase in the level of nerolidol in garlic (*Allium sativum* L.) tissues in response to fungal attack by *Sclerotium cepivorum* [84]. Based on the number of studies reported on the anti-fungal activity of *trans*-nerolidol, it could be suggested that *trans*-nerolidol is a good candidate for the development of anti-fungal drugs.

### 6.5. Anti-Parasitic Activity

Parasitic diseases such as malaria, leishmaniasis, sleeping sickness and Chagas’ disease continue to affect hundreds of millions of people around the world with a majority of them living in tropical regions [123]. However, most of them live in countries where the prospects of any financial return on investment are too low to support market-driven drug discovery and development of new drugs on parasitic diseases. Moreover, the emergence of parasites resistant to current anti-parasitic drugs thwarts the effort in treating the parasitic diseases. All these challenges underline the importance of plant EO as potential novel anti-parasitic agents [89,124].

#### 6.5.1. Anti-Leishmaniasis

Leishmaniasis is a vector-borne infection caused by protozoan parasites from the genus of *Leishmania*. Leishmaniasis affects approximately 350 million people in 88 tropical and subtropical countries. The clinical syndromes and manifestations of leishmaniasis vary widely but are often divided into the three clinically distinct syndromes, the visceral leishmaniasis, cutaneous leishmaniasis (CL), and mucosal leishmaniasis (ML), depending on the parasite species and the host’s immune response [125]. CL has affected mankind for centuries, mainly affecting the skin or mucous membranes and is distinguished by the presence of ulcerative skin lesions. On the other hand, VL is fatal if left untreated and the cutaneous forms are disfiguring and mutilating. Although pentavalent antimonials are still widely used to treat leishmaniasis, they are toxic, poorly tolerated and become increasingly ineffective to cure drug-resistant parasites [126]. Therefore, the search of alternative drugs continues.

Recently, *trans*-nerolidol purified from the leaf EO of *Baccharis dracunculifolia* DC has been found to mediate strong anti-leishmanial activity against promastigotes of *Leishmania* (*L.*) *donovani* with an IC_50_ and IC_90_ values of 42 and 85 µg/mL, respectively [8]. Besides this study, nerolidol also exhibited anti-leishmaniasis activity by inhibiting the growth of *L. amazonensis*, *L. braziliensis*, and *L. chagasi* promastigotes and *L. amazonensis* amastigotes with *in vitro* IC_50_ of 85, 74, 75, and 67 µM, respectively. Moreover, *L. amazonensis*-infected macrophages treated with 100 µM nerolidol resulted in 95% reduction in the rate of infection. Arruda *et al.* suggested that nerolidol at 30 μM mediated anti-leishmaniasis activity through the inhibition of isoprenoid biosynthesis in *L. amazonensis*, as demonstrated by the reduced incorporation of [2-^14^C] mevalonic acid or [1-^14^C] acetic acid precursors into dolichol, ergosterol and ubiquinone in the mevalonate pathway [86]. However, nerolidol did not reduce the incorporation of [1(*n*)-^3^H] farnesyl pyrophosphate into dolichol and ergosterol, suggesting that nerolidol could be an inhibitor at the early step in the mevalonate pathway [86]. Previously, the inhibition of isoprenoid biosynthesis pathway was shown to result in the arrest of development of *Plasmodium falciparum* during the intraerythrocytic stages [127]. Marques *et al.* have also observed similar growth inhibition of promastigotes of *L. amazonensis* after being treated with *trans*-nerolidol purified from the leaves of *Piper claussenianum* (Miq.) C. DC., Piperaceae [9]. *Trans*-nerolidol was also found to induce (1) a significant inhibition (62.17%) on the arginase activity of *L. amazonensis* and (2) an increase in the production of nitric oxide (NO) in *L. amazonensis*-infected macrophages. These results indicated that *trans*-nerolidol was able to interfere with parasite-host cell interaction, thus reducing the percentage of infected cells. Another study conducted by Camargos *et al.* have shown that through electron paramagnetic resonance (EPR) spectroscopy, nerolidol was able to increase the molecular dynamics of the lipid component in the *Leishmania* plasma membrane at IC_50_ of 0.008 µM [87]. This could be possibly due to the insertion of nerolidol into the lipid bilayer that act as spacers to increase the fluidity of membranes since nerolidol has high hydrophobicity, thus causing major reorganization in cell membranes [128]. Subsequently, this will lead to an increase in the overall molecular dynamics of the membrane, causing leakage of cytoplasmic content and eventually the death of *Leishmania* cells.

#### 6.5.2. Anti-Trypanosomal Activity

Trypanosomiasis, also known as sleeping sickness, is caused by protozoan parasites of African trypanosomes (e.g., *Trypanosoma brucei* subspecies) and is fatal if left untreated. Its symptoms include swollen lymph nodes, fever, extreme fatigue and rash. *Trans*-nerolidol purified from the aerial part of *Leonotis ocymifolia* (Burm.f.) Iwarsson and leaves of *Strychnos spinosa* Lam. showed anti-trypanosomal activity with IC_50_ of 15.78 µg/mL and 1.7 µg/mL, respectively on bloodstream forms of *T. brucei brucei* [27,35]. Mohd-Shukri *et al.* conducted an in-depth study about the effects of nerolidol (containing the mixture of ±40% *cis*-nerolidol and ±55% of *trans*-nerolidol) compared to a positive control, berenil (a standard anti-trypanosomal drug) on the morphological changes of a protozoan parasite *Trypanosoma evansi* in mice by using light and electron microscopy [85]. Berenil elicited immediate adverse morphological changes after 2–3 h post-treatment as demonstrated by stiffening and tapering at both ends of the parasite as well as distorted flagella and loss of undulating membranes. On the other hand, nerolidol only induced adverse morphological changes beginning from 23rd to 25th day post-treatment when the parasites became stiff, lost their undulating membrane. At the 27th day post-treatment, total disfigurement was observed, indicating that nerolidol exhibited promising trypanosomatidal activity against the morphology of *T. evansi* in mice. 

#### 6.5.3. Anti-Schistosomal Activity

Schistosomiasis is caused by a trematode blood fluke of the genus *Schistosoma* and is one of the most significantly neglected tropical diseases in the world [129]. Schistosome transmission involves the contamination of water by faeces or urine containing eggs with a specific freshwater snail as intermediate host, followed by human contact with water inhabited by the freshwater snail [130]. Its acute symptoms include fever, urticaria, diarrhea and eosinophilia. However, schistosomiasis, if left untreated, can progress to its chronic stage, leading to inflammatory and obstructive disease in the urinary system (*S. haematobium*) or intestinal disease, hepatosplenic inflammation, and liver fibrosis [130]. According to a study by Parreira *et al.*, the EO of *Baccharis dracunculifolia* DC. (Asteraceae) possessed high schistosomicidal activity since all pairs of *Schistosoma mansoni* adult worms were dead after 24 h incubation with the EO at concentrations of 10, 50, and 100 µg/mL [8]. However, *trans*-nerolidol did not display any significant schistosomicidal activity in the tested assays with the concentration ranging from 10 to 100 µM. In contrast, another experiment conducted by Silva *et al.* have revealed that nerolidol in the form of *cis*- and *trans*-nerolidol racemic mixture (1:1) exerted anti-schistosomal activity by reducing worm motor activity and causing the death of all male and female schistosomes of *Schistosoma mansoni* at concentrations of 31.2 and 62.5 µM, respectively [88]. The differences between these two results could be due to the fact that *trans*-nerolidol isomer is less active than the racemic mixture of *cis*- and *trans*-nerolidol [88]. The study also found that nerolidol induced (1) severe tegumental damage in adult schistosomes and (2) alterations on the tubercles of male parasites in a concentration-dependent manner. With the available findings, a mixture of *cis*- and *trans*-nerolidol was shown to be a promising candidate to treat schistosomiasis.

#### 6.5.4. Anti-Malarial Activity

Malaria is an infection caused by the protozoan parasites belonging to the genus of *Plasmodium* and is transmitted via the bite of *Anopheles* mosquito [131]. Its symptoms are fever, headache, vomiting, sweating and fatigue. If left untreated, it can cause organ failure, abnormal blood coagulation and ultimately death. According to a study conducted by Lopes *et al.*, nerolidol was found to exhibit strong anti-malarial activity since treatment of *Plasmodium falciparum* with 100 mg/mL of nerolidol extracted from the leaf EO of *Virola surinamensis* (Rol. ex Rottb.) Warb. for 48 h resulted in 100% of the inhibition on the development of young trophozoite to the schizont stage without pigment formation [29]. Similarly, nerolidol (23.7%), which is one of the major volatile components extracted from inflorescences oil of *Piper claussenianum* (Miq.) C. DC., has been demonstrated to exert anti-malarial activity with IC_50_ of 11.1 μg/mL whereas the crude oil of *P. claussenianum* showed IC_50_ of 7.9 μg/mL [89]. The study suggested that nerolidol may exert the inhibition of glycoprotein biosynthesis by repressing the biosynthesis of N-glycoproteins that are otherwise observable in *P. falciparum* mainly at the ring and young trophozoite stages of the intra-erythrocytic cycles [29]. Besides this mechanism of action, another study conducted by Rodrigues Goulart *et al.* has shown that the nerolidol inhibited the biosynthesis of the isoprenoid chain attached to the benzoquinone ring in the intraethryocytic stages of *Plasmodium falciparum* [90]. It was evidenced that nerolidol interfered with isoprenoid biosynthesis of apicoplast by disrupting the elongation of isoprenic chains via inhibition of isoprenyl diphosphate synthases, an enzyme that is responsible for the formation of isoprenoid compounds such as dolichols. Beside isoprenyl diphosphate synthase, nerolidol also inhibited the enzyme octaprenyl phosphate/phytoene synthase which is localized in the cytoplasm and also in mitochondria at the intra-erythrocytic stages of *P. falciparum* [132]. Moreover, treatment with nerolidol at doses 2.2 times below the IC_50_ of 0.12 μM was shown to inhibit the production of isoprenic chain attached to coenzyme Q at all intraerythrocytic stages of *P. falciparum* [91]. These findings indicated that nerolidol possesses strong anti-malarial activity by inhibiting the development of the intraerythrocytic stages of the parasites.

Besides displaying anti-malarial activity alone, nerolidol has also been found to exhibit a synergistic effect with either fosmidomycin or squalestatin against malarial parasites. The combination of nerolidol with either fosmidomycin or squalestatin resulted in strong supra-additive (the sum of the fractions of IC_50_ of <1) interaction in mediating inhibition of plasmodial isoprenoid pathway against *P. falciparum* with strong combinatorial IC_50_ of 0.57 and 0.54 μM respectively [92]. 

#### 6.5.5. Other Anti-Parasitic Activities

Nerolidol demonstrated strong nematicidal activity against a nematode, *Caenorhabditis elegans* with its LC_50_ value of 12 µg/mL as well as 74.0% mortality at 50 µg/mL [94]. Besides nematicidal activity, nerolidol (a mixture of *cis*- and *trans*-nerolidol) inhibited the *in vitro* growth of four *Babesia* species with IC_50_ values of 21 ± 1, 29.6 ± 3, 26.9 ± 2, and 23.1 ± 1 µM for *B. bovis*, *B. bigemina, B. ovata,* and *B. caballi*, respectively. This anti-parasitic activity could be due to inhibition of the isoprenoid pathway by nerolidol by a similar mechanism similar to that found with *Plasmodium falciparum* [93]. Nerolidol was also found to be the most active compound among the tested sesquiterpenes (nerolidol, farnesol and elemol) that caused the death of nematodes, L_3_ larvae of *Anisakis simplex* type I with the mortality at 4 hours of 100% at the concentrations of 31.5 and 62.5 µg/mL [95]. Moreover, only 20% of nerolidol-treated rats were affected by gastric wall lesions caused by *Anisakis* larvae in comparison to 86% of the control rats [95].

### 6.6. Insect Repellent Activity

There is a growing concern about the usage of current commercial synthetic insecticides due to the increasing difficulty in the management of pesticide resistance [133]. For this reason, the researchers have focused on research of EOs that have been traditionally used as repellants. Studies in several countries have shown that certain plant EOs are effective not only in repelling insects, but have contact and fumigant insecticidal activity against specific pests without harmful side-effects to humans and animals [134,135]. 

The combination of nerolidol and linalool (that are purified from EO of *Capparis tomentosa* fresh leaves) showed significant repellence activity against maize weevil *Sitophilus zeamais* at all tested doses (0.002, 0.02, 0.2 and 2 µL) [30]. In another study, *trans*-nerolidol derived from the EO of Siam-wood (*Fokienia hodginsii* (Dunn) A.Henry & H H.Thomas) was shown to possess insecticidal activity with LD_50_ value at 0.17 µmol/fly [51]. 

A mixture of nerolidol and tea tree oil with a ratio of 2:1 (tea tree oil 0.5% plus nerolidol 1%) was shown to exert insecticidal and ovicidal activity against *Pediculus capitis* (head lice) and its eggs [96]. Besides, nerolidol purified from the seeds of *Magnolia denudata* Desr. also showed larvacidal activity against third-instar larvae of insecticide-susceptible *Culex pipiens pallens* and *Aedes aegypti* as well as the wild *Aedes albopictus* and *Anopheles sinensis* with lethal dose (LD)_50_ values of 9.84, 13.85, 16.34 and 20.84 mg/L respectively [47]. Similarly, *trans*-nerolidol, which is one of the components of EO from the leaves of *Melaleuca quinquenervia* (Cav.) S.T.Blake, at its concentration of 0.1 mg/mL exhibited strong larvicidal activity with ≥ 95% mortality against *Aedes aegypti* [36]. 

Meanwhile, the *trans*-nerolidol that was purified from the leaves of *Piper aduncum* L. possessed strong acaricidal activity due to its highest repellency of 83.2% ± 0.59% compared to α-humulene (73.3% ± 0.83%) and β-caryophyllene (70.7% ± 0.88%) against the two-spotted spider mite, *Tetranychus urticae* Koch that causes damage to many agricultural crops [37]. The EO of aerial parts of *Baccharis dracunculifolia* DC. containing nerolidol as one of the major components was discovered to demonstrate strong acaricidal activity by causing 100% mortality of *Rhipicephalus microplus* larvae (cattle tick that infests cattle) at 20.0 mg/mL [97]. Meanwhile, a 100% mortality of *Rhipicephalus microplus* larvae was achieved at a lower concentration of nerolidol (15.0mg/mL). The study also demonstrated that nerolidol reduced the quality of the egg and larval hatching rate with increasing concentration from 20 to 50mg/mL [97].

### 6.7. Anti-Ulcer Activity

Gastric ulcer affects thousands of people around the world and is known to be caused by an imbalance between aggressive (acid, pepsin) and protective factor (secretion and action of mucus and bicarbonate) in the stomach [98]. It is induced by several factors, such as stress, smoking, nutritional deficiencies and ingestion of non-steroidal anti-inflammatory drugs (NSAIDs). The current therapy for ulcers usually involves the use of histamine H_2_-antagonists, proton pump inhibitors and anti-muscarinics for the inhibition of gastric acid secretion. However, these drugs pose severe side-effects, particularly hypersensitivity, arrhythmia and impotence [136]. With this in mind, plant EOs have recently been exploited as they have been shown to produce promising results for alternative therapies to treat gastric ulcers with lesser side-effects. 

A study has been conducted by Klopell *et al.* on the anti-ulcer property of nerolidol using different experimental models such as ethanol-, indomethacin- and stress-induced ulceration in rat [98]. In the stress-induced ulceration model of experiment, nerolidol treatment at 50, 250 and 500 mg/k caused a significant reduction in the ulcerative lesion index (ULI) by 41.22, 51.31 and 56.57, respectively when compared to the control group animals. With regard to the ethanol-induced ulceration model of experiment, treatment with nerolidol at 250 and 500 mg/kg significantly inhibited the formation of ulcer at 52.63% and 87.63%, respectively as compared to the control group. On the other hand, indomethacin-induced ulceration model of experiment, the treatment at 250 and 500 mg/kg of nerolidol had significantly inhibited the gastric ulcer for 51.02% and 46.93%. These findings indicate that nerolidol could be used as an active component in gastroprotective and anti-ulcer treatments.

### 6.8. Skin Penetration Enhancer Activity

Transdermal delivery has gained a lot of attention as an attractive alternative route to intravenous and oral drug delivery systems [137]. However, the application of transdermal delivery is limited by poor drug permeability as the stratum corneum plays as a rate-limiting lipophilic barrier against the uptake of chemical and biological agents [138]. Therefore, terpenes are often used as topical skin penetration enhancers due to their wide range of physicochemical properties such as low cutaneous irritancy and good toxicological profile as well as adsorption enhancement ability [139]. 

Nerolidol has been found to be a potent skin penetration enhancer. It was found to increase the diffusion rate by over 20-fold for transdermal delivery of several drugs especially on 5-fluorouracil [99]. This high permeation-enhancing ability was attributed to the structure of nerolidol which is suitable for the alignment within lipid lamellae of the stratum corneum in order to disrupt the organization of stratum corneum. This view has been further supported by Prasanthi and Lakshmi [100] who reported that nerolidol with highest lipophilicity (log P = 5.36 ± 0.38) have the highest enhancement effect with its rank of order of nerolidol > farnesol > limonene > linalool > geraniol > carvone > fenchone > menthol in facilitating transdermal delivery of alfuzosin hydrochloride. Similarly, nerolidol has the highest permeation enhancing ability with a 3.2-fold increase in permeation of selegiline hydrochloride across the rat skin, followed by carvone (2.8-fold increase) and anethole (2.6-fold increase) [101]. Another study conducted by El-Kattan *et al.* has shown that nerolidol was the most effective percutaneous permeation enhancer for four model drugs (nicardipine hydrochloride, hydrocortisone, carbamazepine, and tamoxifen) when compared to other terpenes (fenchone, thymol, nerolidol and d-limonene) [102]. 

### 6.9. Anti-Nociceptive and Anti-Inflammatory Activity

Pain is an unpleasant sensation and emotional experience that is associated with actual or potential tissue damage [140]. On the other hand, the stimulation of nociception is associated with the detection of real tissue injury or a potentially damaging event by nociceptors as a stimuli (transduction) followed by its transmission of encoded information to the brain [141]. Under normal circumstances, the presence of an injury activates the inflammatory response as follows: firstly the inflammatory mediators are released from damaged cells such as ions (K^+^, H^+^), bradykinin, histamine, 5-hydroxytryptamine (5-HT), ATP and nitric oxide. Subsequent activation of arachidonic acid pathway leads to the production of prostanoids and leukotrienes that would then lead to the release of more inflammatory mediators such as cytokines and growth factors. These mediators will ultimately activate peripheral nociceptors directly, resulting in spontaneous pain. Beside this action, they also act to convert responses of primary afferent neurons to subsequent stimuli (peripheral sensitization) to be transmitted to the brain. Due to exacerbated physiological response, chronic exposure to pain is very harmful to an individual as it can cause organ damage and ultimately death, if left untreated [142].

Non-steroid anti-inflammatory drugs (NSAIDs) are well-known analgesic drugs that act to reduce inflammation and pain by acting as an inhibitor of cyclooxygenases (COXs) in the arachidonic acid pathway. However, one major disadvantage of administration of NSAIDs is their serious side-effects such as significant gastrointestinal upset, gastritis, ulceration, hemorrhage, and even death [143]. In order to address this issue, EOs extracted from various medicinal plants have been increasingly explored as alternative traditional medicines to treat inflammation and pain without posing harmful side-effects.

Pinheiro *et al.* investigated the anti-nociceptive and anti-inflammatory effects of EO of *Peperomia serpens* (Sw.) Loudon in rodents [38]. The EO has been found to possess anti-inflammatory and anti-nociceptive activities which could possibly be mediated by one of its major compounds, *trans*-nerolidol (38.0%). In a similar experiment, Lima *et al.* reported anti-nociceptive and anti-inflammatory activities of the EO of the aerial parts of *Piper aleyreanum* C. DC which is attributable to the presence of *trans*-nerolidol (1.2%) [10]. In order to strengthen the findings, Fonsêca *et al.* investigated the anti-nociceptive and anti-inflammatory activities of nerolidol using mouse models of pain [103]. The study found that nerolidol has no effect on the locomotor activity. Meanwhile, anti-nociceptive activity was evaluated via acetic acid-induced writhing and the formalin tests. The results demonstrated that oral administration of nerolidol was able to cause lesser acetic acid-induced abdominal contractions and also inhibition in paw licking behavior in the respective tests when compared to the control group (Table 4). These results implied that nerolidol modulates its effect on neuropathic pain and inflammatory processes as demonstrated by the formalin test [103]. However, the anti-nociceptive effect of nerolidol did not involve the thermal stimulation of centrally mediated nociception as shown by the negative hot-plate test result. In addition, the researchers also further elucidated the possible anti-nociceptive mechanisms of nerolidol by examining its effects on the parameters GABAergic system, opioidergic and ATP-sensitive K+ channels. The results have shown a positive association of nerolidol with the GABAergic system but not with opioidergic or ATP-sensitive K+ channels, implying that the anti-nociceptive activtity of nerolidol is mediated through GABA_A_ receptors [103]. In order to evaluate the anti-inflammatory activity of nerolidol, carrageenan-induced paw edema was used as a model of inflammation. It was found that nerolidol exhibited inhibitory effect on inflammation. Further investigation of carrageenan-induced peritonitis model revealed that nerolidol decreased the levels of polymorphonuclear cells and tumor necrosis factor (TNF-α) in peritoneal lavage as well as interleukin 1 beta (IL-1b) in LPS-stimulated, peritoneal macrophages (Table 4) [103]. Taken these results together, nerolidol has been shown to demonstrate promising analgesic and anti-inflammatory activities.

### 6.10. Anti-Cancer and Anti-Tumor Activity

Cancer is one of the most alarming causes of death, with an estimated over six million deaths have been reported around the world annually. It is a multifactorial disease that leads to uncontrolled growth and invasion of abnormal cells, ultimately leading to the formation of tumor. Chemotherapy, radiosurgery and surgery are some of the effective treatments against various type of tumors. Despite that, these treatments still pose many side-effects that lead to acute and chronic organ damage such as bone marrow suppression, hepatic, pulmonary, cardiac, renal and gastrointestinal toxicities [144,145]. Moreover, the development of drug resistance in tumors have also been reported during the courses of chemotherapy. This may be due to the occurrence of mutations in the tumor cells that negates the apoptotic pathway during cancer treatment [146]. These drawbacks of chemotherapy treatment have urged researchers to find alternative treatments without harming the growth of normal cells and triggering anti-tumor drug resistance. Among the alternative approaches, plant phytochemicals have been recently explored for their possible beneficial (anti-proliferative and cytotoxic) effects on cancer cells *in vitro* or *in vivo* models [147,148].

#### 6.10.1. *In Vitro* Studies

Several studies have shown anti-tumor properties of nerolidol on cancer cell lines. A study conducted by Ryabchenko *et al.* has demonstrated strong anti-tumor effects of nerolidol (a combination of *cis*-nerolidol 40.7%, *trans*-nerolidol 58.3%, *cis*-dihydronerolidol 0.4% and *trans*-dihydronerolidol). It was found to reduce the viability of HeLa cells at its concentration (CC_50_) of less than 5 µM (1.5 ± 0.7 µM) [104]. Beside this study, nerolidol (isomer not specified), which is one of the ten major compounds found in the green tea flavor, exhibited strong cytotoxicity effect with an IC_50_ value of 2.96 and 3.02 µg/mL against BT-20 breast carcinoma cells and HeLa cells, indicating that nerolidol potentially exhibit strong anti-cancer activity against the two tumor cell lines [106]. This has been further supported by the fact that *trans*-nerolidol, which was purified from the leaf EO of *Zornia brasiliensis* Vogel, had a strong cytotoxicity activity against cancer cell lines such as B16-F10 (mouse melanoma), HepG2 (human hepatocellular carcinoma), HL-60 (human promyelocytic leukemia) and K562 (human chronic myelocytic leukemia) with IC_50_ values of >25, >25, 21.99 and 17.58 µg/mL, respectively using Alamar blue assay [39]. The study also reported no cytotoxicity effect of *trans*-nerolidol on non-tumor cells, particularly the peripheral blood mononuclear cells (PBMCs) [39]. Another study conducted by Boris *et al.* have shown that *cis*-nerolidol exhibited the strongest cytotoxic activity (16.5 ± 6.7 μM) against HeLa cells among other sesquiterpene alcohols such as α-bisabolol, cedrol, patchoulol, and santalol [105]. Beside green tea, *trans*-nerolidol extracted from leaf EO of *Comptonia peregrina* (L.) Coult. has been found to induce strong cytotoxic effect against human lung carcinoma A-549 and colon adenocarcinoma DLD-1 cell lines with IC_50_ values of 6.4 ± 0.4 and 5.8 ± 0.4 µg/mL, respectively [149]. Another anti-proliferative study conducted by Ambrož *et al.* has shown that *trans*-nerolidol potentiated the action of doxorubicin, an anticancer drug, by increasing killing of CaCo-2 cancer cells [40]. The study also reported no cytotoxicity effect of *trans*-nerolidol on rat hepatocytes that serve as non-tumor cells [145]. Based on the literature, it can be suggested nerolidol is a good candidate for the development of anticancer agent that selectively targets specific cancerous cells with no cytotoxicity towards PBMCs and rat hepatocytes. 

When examining the cytotoxic effect of nerolidol with regard to the apoptotic pathway, the combination of two acylic isoprenoids, farnesol and nerolidol was found to suppress the proliferation of human HL-60 acute promyelocytic leukemia (HL-60) cells by 20%, which was slightly synergistic (slightly exceeding the 13% suppression obtained from the sum of both compounds); farnesol isomers (2.5 µmol/L) and nerolidol (5 µmol/L) individually suppressed the proliferation of HL-60 cells by 4% and 9%, respectively [108]. The mechanism of action involved prolonging the cell cycle arrest of HL-60 cells at the G_0_-G_1_/S interphase and lead to apoptotic cell death. Another study conducted by Hanušová *et al.* focused on the anti-proliferative effect of EO from leaves of *Myrica rubra* (Lour.) Siebold & Zucc. (MEO) and its major compound, *trans*-nerolidol on the adhesion, expression of adhesion molecules (ICAM-1; *E*-cadherin; β-catenin and apoptotic molecules (NF-κB, caspases) in colorectal cancer cell line HT29 [147]. The study showed that only the MEO reduced the cell adhesion to collagen. Meanwhile, both MEO and *trans*-nerolidol (30 µg/mL) significantly suppressed cell adhesion of HT29 cells in the presence of TNFα and it was suggested due to the down-regulation of ICAM-1 [109]. Besides, *trans*-nerolidol (30 µg/mL) also significantly increased the expression of E-cadherin [109], a cell adhesion molecule that mediates the suppression of epithelial cell tumor invasiveness [150]. MEO and *trans*-nerolidol were also found to decrease the phosphorylation of NF-κB and activate caspases activity in TNFα-induced HT29, thereby leading to apoptosis of cancer cells [109].

#### 6.10.2. *In Vivo* Studies

In animal studies, nerolidol has been found to possess strong anti-tumor activity by inhibiting the intestinal carcinogenesis induced by azoxymethane (15 mg/kg body weight) administered twice per week for a duration of three weeks in male F344 rats [107]. The result showed the reduction of incidence of intestinal neoplasia from 82% to 33% in rats fed with nerolidol. Moreover, the number of tumors/rat was reduced from 1.5 to 0.7 in rats fed with nerolidol. The improvement of intestinal carcinogenesis was possibly due to the modulatory effect nerolidol on protein prenylation, a post-translational process that is required to cause cancer [151]. Another study conducted by Costa *et al.* has demonstrated that the EO of *Zornia brasiliensis* Vogel leaf at dose of 100 mg/kg containing *trans*-nerolidol as major constituent reduced the weight of tumor in mice injected with B16-F10 melanoma by 38.61% when compared to the untreated group [39]. 

## 7. Pharmacokinetic Studies

Although there is an increasing popularity of herbal medicines and essential oils, they are not properly screened for purity and potency which may raise serious questions regarding their possible herb-drug interaction with conventional medicine that may cause serious adverse effects on human health [152]. In order to address this issue, more pharmacokinetic and toxicological research have been conducted to examine the efficacy and safety of essential oils.

### 7.1. In Vitro Studies

In order to determine the recovery of nerolidol, *Saccharomyces cerevisiae* prototrophic haploid strain IWD72 was incubated in YEPD medium comprised of yeast extract, bacteriological peptone, glucose, adenine, and uracil. Nerolidol (100 μg/mL) was subsequently added to the 50 mL bacteria culture in YEPD medium. The aerobic culture cells were harvested by centrifugation after 24 h, and the cells were collected for the recovery of nerolidol. Residual nerolidol recovered at 24 h was 79.0 µg/mL [14,153].

### 7.2. In Vivo Studies

Rats were fed 20 and 40 mg of nerolidol that were mixed with 1 mL of cottonseed oil and 30–35 mL of evaporated milk per day, for eight days. The average daily excretion on the 1st to 4th and 4th to 8th day was monitored. Rats fed with 20 mg/day of nerolidol excreted 0.3 and 0.7 mg of nerolidol per day with a maximum average of 0.9 mg. On the other hand, the rats fed with 40 mg/day of nerolidol excreted 1.0 and 1.6 mg per day with a maximum average of 2.1 mg [14,154]. 

Based on a recent *in vivo* pharmacokinetic study conducted by Saito *et al.*, nerolidol (*cis*-*/trans*-nerolidol, 1:3; *w*/*w*) was quantitatively determined in mouse plasma using GC-MS method [22]. Three BALB/c mice weighing 20 ± 2 g were firstly fed orally with a single oral dose of 1000 mg/kg of nerolidol. Blood samples were then taken at 30 min, 1, 2, 3, 4, 5, 6, 8, and 12 h after oral administration, followed by separation of plasma from blood via centrifugation and GC-MS analysis. The level of nerolidol was observed in the plasma with its maximum concentration of ∼0.27 ± 0.07 μg/mL within 30 min after oral administration and remained constant for up to 3 h after administration, reaching a maximum concentration of ~0.35 ± 0.05 μg/mL after 6 h of administration. The concentration of nerolidol in plasma after 6 hours was twice the IC_50_ concentration of *in vitro* nerolidol administration on *P. falciparum* (0.169 μg/mL) [155]. Moreover, this maximum concentration was detected to be ~1460 times lower than the concentration required to induce 50% hemolysis (∼511 μg/mL). The concentration of nerolidol in plasma decreased to near zero 12 h after oral administration. These results indicated that the maximum concentration in mouse plasma after oral administration was below the hemolytic concentration, thus the safe oral dose is up to 1000 mg/kg. 

In another recent study, He *et al.* utilized LC-MS instead of GC-MS method to quantitatively determine the *in vivo* pharmacokinetics of nerolidol (*cis*-/*trans*-nerolidol, 2:3) in rat plasma [25]. Sprague-Dawley rats weighing 250–300 g were firstly administered only once with 25 mg/kg of nerolidol via intraperitoneal injection. Blood samples were then collected at 10, 20, 30, 60, 90, 120, 240 and 360 min after injection, followed by separation of plasma from blood via centrifugation. Through the LC-MS analysis, they revealed that the maximum concentration of nerolidol observed in rat plasma was 8.30 ± 1.07 μg/mL at 20 min after single intraperitoneal injection. The concentration of nerolidol in plasma decreased to near zero two hours after intraperitoneal injection.

A comparative pharmacokinetic study between work by Saito *et al.* [22] and He *et al.* [25] revealed that the oral administration of nerolidol exhibited lower peak plasma concentration than that of intraperitoneal administration (Table 5). This is because orally administered nerolidol must first undergo first-pass effect by which it must pass through the intestinal wall and then to the portal circulation and liver [157]. As a result, a portion of the oral dose of nerolidol was lost during the first pass metabolism in the liver, thus contributing to low bioavailability as compared to that of intraperitoneal injection [158]. Another key point of the pharmacokinetic comparison between the two studies is that the human equivalent doses (HEDs) of both administration calculated in Table 5 can be used as appropriate starting doses of nerolidol in human clinical trials.

### 7.3. Toxicological Studies

Despite the long history of therapeutic uses of EOs and its general acknowledgment by the public, the assumption that the HEDs can be used for the first clinical trial has however seldom been verified, thus raising the concern of safety issues pertaining the usage of EOs [159]. For that reason, toxicological screenings are required to assess the potential toxicities induced by the EOs.

#### 7.3.1. Acute Toxicity

The dermal LD_50_ values of nerolidol (isomer not specified) in rabbit was found to be higher than 2000 mg/kg body weight, thus indicating low acute toxicity via transdermal route [13]. In terms of oral administration, the oral LD_50_ values of nerolidol in rats and mice were higher than 2000 mg/kg body weight (>5000 and 9976 mg/kg body weight, respectively), indicating low acute toxicity via oral route [13].

#### 7.3.2. Skin Irritation and Sensitization Studies

In human studies, 4% nerolidol (isomer not specified) administration in a pre-test for a maximization study with single occlusive application for 48 h did not cause skin irritation [13]. On the other hand, the application of undiluted nerolidol (isomer not specified) on intact and abraded skin caused well-defined erythema or slight edema in rabbits after 48 h, whereas 5% nerolidol in diethylphthalate caused very slight edema in one animal and was cleared after 48 h [13]. With regard to skin sensitization, human studies have shown that nerolidol (isomer not specified) (4%) did not cause any positive reactions in maximization tests or repeated insult patch tests. In animal studies, the administration of nerolidol in guinea pigs resulted in weak reactions in two adjuvant tests with concentrations of 3% and 10% [160,161]. However, no cross-sensitization was observed when guinea pigs induced with farnesyl acetate were cross-sensitized with nerolidol.

#### 7.3.3. Mucous Membrane Irritation

No human data is available for mucous membrane irritation studies [13]. However, in animal studies, undiluted nerolidol initially caused very slight redness, but was cleared by 2 h. On the other hand, 5% nerolidol in DEP poses no mucous membrane irritation [13]. 

#### 7.3.4. Phototoxicity and Photoallergenicity

UV spectra for nerolidol indicated that it did not absorb UVB light (290–320 nm) [162]. However, nerolidol peaked at the UVC range (220–240 nm) with a very slight absorption at 250–300 nm range, thus do not possibly induce phototoxicity or photoallergy under the current conditions of use as fragrance ingredients [13].

#### 7.3.5. Reproductive and Developmental Toxicity

In order to investigate the development of fetal epidermal permeability barrier *in vitro*, the activators of the receptors for vitamin D_3_ and retinoids, and of the peroxisome proliferator activated receptors (PPARs) and the farnesoid X-activated receptor (FXR) were examined. Sprague-Dawley rats were firstly impregnated (plug date = day 0) in order for the skin of 17-day old fetus to be cultured for the measurement of barrier function. The effect of activators of FXR, isoprenoid precursors and metabolites on the development of epidermal barrier was monitored. Explants were incubated in the presence of 100 μM nerolidol for two days. Full-thickness flank skin was excised from fetal rats for skin analysis with light and electron microscopy. The results have shown that nerolidol did not activate the FXR, thus did not alter the epidermal barrier during skin development [14,163].

#### 7.3.6. Cytotoxicity and Genotoxicity

##### 7.3.6.1. In Vitro *Studies*

Due to nerolidol being used as a potent skin permeation enhancer with low toxicity, a number of studies were conducted on the toxicological properties of nerolidol. Mendanha *et al.* compared the hemolytic and toxic effects of nerolidol and various monoterpenes on fibroblast cells as well as their effect on erythrocyte membrane fluidity [155]. By using the 3T3 NRU assay to evaluate the cytotoxicity of nerolidol and various monoterpenes (α-terpineol, l-(−)-carvone, (+)-limonene, l-menthone, d,l-menthol, pulegone and 1,8-cineole) on fibroblast cells, nerolidol was found to be the most cytotoxic with its IC_50_ value of 0.06 ± 0.01 mM. Besides, nerolidol caused 50% hemolysis at 2.3 ± 0.8 mM and induced a significant increase in the fluidity of erythrocyte membrane at 2.5 × 10^9^ molecules/cell, indicating that the nerolidol possessed the highest hemolytic effect on erythrocyte membrane fluidity when compared to terpenes. In addition, electron paramagnetic resonance (EPR) spectroscopy of the spin label 5-doxyl stearic acid (5-DSA) was used to investigate the effect of terpenes on membrane fluidity in erythrocyte and fibroblast cells. Nerolidol was found to be more potent than terpenes that caused an increase in the membrane fluidity. The results implied that nerolidol was able to increase membrane fluidity but also had increased ability to disrupt the membrane and had higher cytotoxic potential.

Ferreira *et al.* further examined the toxicity of nerolidol (a racemic mixture of *cis*- and *trans*-isomers) (1:1) on mitochondrial and cellular energetics in *in vivo* model using Wister rat liver mitochondria and *in vitro* model using HepG2 (human hepatocellular liver carcinoma) cells [12]. In the *in vitro* study, nerolidol exertes hepatic cell cytotoxicity due to a decrease in ATP/ADP levels by negatively interfering with hepatic mitochondrial bioenergetics in concentrations lower than 2.4 μM. Consequently, nerolidol induced cell arrest and cell death which is possibly due to the inhibition of F_0_F_1_-ATP synthase in a concentration-dependent manner. In the *in vivo* study, nerolidol (low doses up to 2.4 μM) induced a decrease in transmembrane electric potential in the mitochondrial membrane isolated from rat liver in a concentration-dependent manner [12]. By decreasing transmembrane electric potential, nerolidol could negatively affect the hepatic mitochondrial bioenergetics as it would cause mitochondrial dysfunction, thus leading to hepatic cell cytotoxicity and eventually cell death [164].

Marques *et al.* investigated the cytotoxicity effect of EO extracted from leaves of *P. claussenianum* and reported no toxicity effect in the fibroblasts nor macrophages cell lines in any concentration tested (ranging from 40 to 0.56 mg/mL) [9]. Similarly, the EO extracted from leaves of *P. claussenianum* did not induce toxicity on the L929 mouse fibroblast cells [78]. Peres *et al.* investigated the cytotoxicity effect of EO purified from *Piper gaudichaudianum* Kunth in which its major compounds were *trans*-nerolidol, α-humulene, (*E*)-caryophyllene and bicyclogermacrene [165]. Although dose-dependent cytotoxicity effect of the EO as well as single-strand DNA breakage were observed in the Chinese hamster lung fibroblast cells (V79 cells), however, no double-strand breaks occurred. Furthermore, the EO induced a significant increase in lipid peroxidation at higher dose. The results indicated that the EO possessed strong cytotoxicity, genotoxic and mutagenic effects which were attributed to the role of nerolidol. Due to this reason, Sperotto *et al.* further investigated the cytotoxic and mutagenic properties of the EO of *P. gaudichaudianum* as well as its major compound *trans*-nerolidol using *Saccharomyces cerevisiae* as a model organism [166]. *P. gaudichaudianum* EO was found to induce cytotoxic effects in the XV185-14c and N123 strains of *S. cerevisiae* but induced mutagenesis only at the *lys1* locus at the highest concentration of 100 µg/mL. On the other hand, nerolidol (a racemic mixture of *cis*- and *trans*-isomers) (1:1) was found to be cytotoxic in XV185-14c at concentration of 25, 50 and 100 µg/mL and did not significantly cause any induction of mutagenicity at the three loci evaluated. Moreover, EO and nerolidol were discovered to generate ROS via DCF-DA probing assay in superoxide dismutase (Sod)-deficient strains. Based on these findings, Sperotto *et al.* [166] claimed that nerolidol shows a weak mutagenic effect but exerts strong cytotoxicity effect which could be attributed to the formation of reactive oxygen species (ROS) and the formation of single-strand breaks. Despite that, more in depth analysis involving molecular techniques could be conducted to elucidate the mechanisms that are responsible for the strong cytotoxic but weak mutagenic effects of nerolidol. Furthermore, toxicogenomics could be an option to be considered in further evaluating the safety of nerolidol. Briefly, toxicogenomic is based on the integration of genomics and toxicology with the aim of studying the toxicity of xenobiotics on the biological systems via global analysis of genome-wide mRNA expression (transcriptomics), protein (proteomics) and metabolite patterns (metabonomics) [167]. Due to its wide usage in the research of plant-based medicinal natural products, particularly in traditional Chinese medicine (TCM) [168], toxicogenomics could perhaps be an effective tool in evaluating the safety of nerolidol at the genomic level to ensure that it is safe for humans. Besides that, more *in vitro* experiments investigating the induction of micronuclei, chromosome aberration or telomere shortening effect of nerolidol may also be performed in order to confirm the genotoxicity status of nerolidol in human or mammalian cells.

##### 7.3.6.2. In Vivo *Studies*

Pículo *et al.* had investigated the genotoxicity and clastogenicity (ability to cause DNA damage by inducing chromosomal aberrations) effects of *trans*-nerolidol in blood and liver cells of 12-week-old male Swiss albino mice (*Mus musculus*) using comet and micronucleus assays respectively [169]. The experiment was based on the cytotoxicity effect analysis by scoring 200 consecutive total polychromatic (PCE) and normochromatic (NCE) erythrocytes (PCE:NCE ratio) in bone marrow cells and no significant decrease in PCEs:NCEs ratios at the three doses tested (250, 500 and 2000 mg kg^−1^) was observed, indicating the absence of cytotoxic effects of *trans*-nerolidol at these doses. Nevertheless, weak genotoxic effects of *trans*-nerolidol in the blood and liver cells were observed and a slight increase in the DNA damage at higher doses. Based on the available studies, it can be deduced that the non-toxic dose of *trans*-nerolidol for animal is up to 2000 mg/kg. Moreover, no DNA damage was observed in the peripheral blood cells and liver of the animals. In order to determine the safety of nerolidol consumption in humans, more clinical trials should be conducted to assess and validate the toxicity and side effects of nerolidol in humans. 

## 8. Conclusions

Nerolidol is one of the common components found in the essential oil of various medicinal plants. A majority of the studies reveal that nerolidol is the major constituent in many plants that accounts for their pharmacological and biological activities such as anti-microbial, anti-parasitic, anti-biofilm, anti-oxidant, anti-nociceptive, anti-inflammatory, anti-ulcer, skin penetration enhancer, insect repellent and anti-cancer properties. Based on pharmacokinetic and toxicological data available, the dosage of nerolidol is considered safe to be translated from animal to clinical studies in order to evaluate its efficacy. Taken all together, nerolidol has a great potential to be used as a new chemical or therapeutic drug in the field of agriculture and medicine, respectively and sufficient baseline information is available for guiding future works and commercial exploitation.

## Figures and Tables

**Table 1:** Synonyms of *cis*- and *trans*-nerolidol.

*Cis*-Nerolidol	*Trans*-Nerolidol
(i) (±)-*cis*-nerolidol	(i) (±)-*trans*-nerolidol
(ii) (6*Z*)-3,7,11-trimethyl-1,6,10-dodecatrien-3-ol	(ii) (6*E*)-3,7,11-trimethyl-1,6,10-dodecatrien-3-ol
(iii) (6*Z*)-3,7,11-trimethyldodeca-1,6,10-trien-3-ol	(iii) (6*E*)-3,7,11-trimethyldodeca-1,6,10-trien-3-ol
(iv) (6*Z*)-nerolidol	(iv) (6*E*)-nerolidol
(v) 1,6,10-dodecatrien-3-ol, 3,7,11-trimethyl-, (6*Z*)-	(v) 1,6,10-dodecatrien-3-ol, 3,7,11-trimethyl-, (6*E*)-
(vi) (*Z*)-nerolidol	(vi) (*E*)-nerolidol

**Table 2:** Plant sources of nerolidol along with its percentage of nerolidol and extraction method.

Plant Part	Type of Nerolidol Found in the Essential Oil	Nerolidol Purified from the Essential Oil of the Respective Plants (%)	Extraction Method	Ref.
Aerial parts	*trans*-nerolidol	(i) *Warionia saharae* ex Benth. & Coss. (23.0%)	Hydrodistillation technique using the Clevenger-type apparatus	[10,26,27,28]
		(ii) *Scutellaria abida* L. ssp. *albida* (9.03%)		
		(iii) *Piper aleyreanum* C. DC (1.2%)		
		(iv) *Leonotis ocymifolia* (Burm.f.) Iwarsson (0.41%)		
Leaf	Nerolidol (n.s.)	(i) *Capparis tomentosa* Lam. (5.14%)	Hydrodistillation technique using the Clevenger-type apparatus	[29,30]
		(ii) *Virola surinamensis* (Rol. ex Rottb.) Warb. (3.0%)		
		*Ginkgo biloba* L. (0.12%)	Molecular distillation at a feed temperature of 60 °C, distillation temperature of 280 °C, feed flow rate of 180 mL per hour, scraper rate of 300 rpm, and operating pressure of 0.1–0.5 Pa	[31]
	*trans*-Nerolidol	(i) *Baccharis dracunculifolia* DC. (33.51%)	Hydrodistillation technique using the Clevenger-type apparatus	[8,9,32,33,34,35,36,37,38,39,40,41,42,43]
		(ii) *Cassia fistula* L. (2.2%)		
		(iii) *Comptonia peregrina* (L.) Coult. (2.11% and 3.43% after 0–30 min fraction and 30–60 min fraction respectively)		
		(iv) *Melaleuca quinquenervia* (Cav.) S.T.Blake (24.19%)		
		(v) *Myrica rubra* (Lour.) Siebold & Zucc. (2%)		
		(vi) *Lantana radula* Sw. (19.0%)		
		(vii) *Peperomia serpens* (Sw.) Loudon (38.0%)		
		(viii) *Piper aduncum* L. (0.2%)		
		(ix) *Piper chaba* Hunter (5.1%)		
		(x) *Piper claussenianum* (Miq.) C. DC. (81.4%)		
		(xi) *Strychnos spinosa* Lam. (0.7%)		
		(xii) *Swinglea glutinosa* (Blanco) Merr. (28.4%)		
		(xiii) *Zanthoxylum hyemale* A.St.-Hil. (51.0%)		
		(xiv) *Zornia brasiliensis* Vogel (48.0%)		
Stem	*trans*-Nerolidol	*Oplopanax horridus* (Sm.) Miq. (54.5%)	Steam distillation using a low pressure system with an external steam source	[44]
Flower	*trans*-Nerolidol	(i) *Achillea millefolium* L. (11.6%–31.9%)	Hydrodistillation technique using the Clevenger-type apparatus	[42,45,46]
		(ii) *Cananga odorata* (Lam.) Hook.f. & Thomson (0.32%)		
		(iii) *Cassia fistula* L. (38.0%)		
Root	*trans*-Nerolidol	*Oplopanax horridus* (Sm.) Miq. (54.6%)	Steam distillation using a low pressure system with an external steam source	[44]
Seed/grain	Nerolidol (n.s.)	*Magnolia denudata* Desr. (2.18%)	Hydrodistillation technique using the Clevenger-type apparatus	[47]
	*trans*-Nerolidol	(i) *Elettaria cardamomum* (L.) Maton (3.6%)	Hydrodistillation technique using the Clevenger-type apparatus	[7,48]
		(ii) *Momordica charantia* L. (61.6%)		
Fruit	*trans*-Nerolidol	*Swinglea glutinosa* (Blanco) Merr. (19.1%)	Hydrodistillation technique using the Clevenger-type apparatus	[43]
Resin	*trans*-Nerolidol	*Canarium schweinfurthii* Engl. (14%)	Hydrodistillation technique using the Clevenger-type apparatus	[49]
Twig/wood	*Trans*-Nerolidol	*Cinnamomum osmophloeum* Kaneh. (1.05%)	Hydrodistillation technique using the Clevenger-type apparatus	[50]
		*Fokienia hodginsii* (Dunn) A.Henry & H H.Thomas (34.8%)	Solid-phase microextraction	[51]
	*cis-*Nerolidol	*Myrocarpus fastigiatus* Allemao (80.0%)	Hydrodistillation technique using the Clevenger-type apparatus	[52]

Key: n.s. = not specified.

**Table 3:** Retention indices of different chromatographic columns of GC and major peaks of mass spectrometry to differentiate *cis*- and *trans*-nerolidol.

Types of Column/Equipment Used	*Cis*-Nerolidol	*Trans*-Nerolidol	Ref.
**(A) Retention time of different chromatographic columns of GC (minutes)**			
(i) A-100 or 154-C column	14	16	[22]
(ii) DB-5 capillary column	n.a.	10.5	[21]
(iii) TR-5MS capillary column	5.87	5.98	[22]
**(B) Retention time of different chromatographic columns of LC (minutes)**			
(i) Hypersil BDS C18 column	11.9	13.1	[25]
**(C) Major peaks of mass spectrometry (MS) (m/z)**			
(i) M-80B gas chromatograph double focusing mass spectrometer	41, 69, 134, 91, 93, 79	69, 41, 93, 43, 71, 55	[55]
(ii) Y2K ion trap (MS) PolarisQ System mass spectrometer	93, 91, 67, 107, 79, 161, 121, 133, 55, 147, 189, 175	93, 121, 67, 107, 79, 161, 136, 55, 189, 148, 175	[22]
**(D) Retention indices of different chromatographic columns of GC**			
(i) HP-101	n.a.	1564	[56]
(ii) HP-20M	n.a.	2009	[56]
(iii) HP-FFAP	n.a.	2055	[56]
(iv) Fused silica capillary column coated with DB-5	n.a.	1564	[29]
(v) OV-101	1533	1549	[55]
(vi) PEG 20M	2028	2035	[55]
(vii) DB-5	1565	1539	[57]
(viii) DB-Wax	2010	2054	[57]
(ix) SPB-1	1543	n.a.	[58]
(x) Dimethylsilicone (DIMS)	1524.4 (a)	1550.1 (a)	[54]
(xi) Dimethylsilicone with 5% phenyl groups (DIMS5P)	1543.6 (a)	1560.9 (a)	[54]
(xii) Polyethylene glycol (PEG)	2007.3 (a)	2036.3 (a)	[54]

Key: (a) = average value; n.a. = not available.

**Table 4:** A summary of pharmacological and biological activities of nerolidol.

Bioactivity	Type of Nerolidol	Plant and Part of Plant Used (If Any)	Target Organism(s)	Screening Assay and Methods Used	Results	Possible Mechanisms of Action	Ref.
Antioxidant activity	*cis*-Nerolidol (Aldrich Chemical Co., Milwaukee, WI, USA)	-	-	DPPH and hydroxyl radical scavenging activity	(i) Exhibited DPPH radical scavenging activity	Mediates antioxidant activities via free radical scavenging activity	[73]
					(ii) Exhibited scavenging activity against hydroxyl radical with IC_50_ = 1.48 mM		
	*cis*-Nerolidol (Sigma-Aldrich, St. Louis, MO, USA)	-	-	Thiobarbituric acid reactive substances (TBARS) assay	(i) Demonstrated 25.60% ± 0.98% malonaldehyde (MDA) reduction in hepatocytes at 1 mM under physiological conditions	Mediates antioxidant activity via lipid peroxidation inhibitory effect	[74]
					(ii) Demonstrated higher MDA reduction with value of 36.50% ± 4.47% at 1 mM in hepatocytes under oxidative stress induced by *tert*-BuOOH		
	Mixture of *cis*- and *trans*-nerolidol (Sigma Chemical Company, St. Louis, MO, USA)	-	-	TBARS assay, nitrite assay, superoxide dismutase (SOD) activity and catalase activity	(i) At doses of 25, 50 and 75 mg/kg of nerolidol caused a significant decrease in lipid peroxidation by 59.97%, 74.79% and 91.31% respectively when compared to negative control	(i) Suggested to prevent oxidation of polyunsaturated fatty acids	
					(ii) At doses of 25, 50 and 75 mg/kg of nerolidol caused a significant decrease in nitrite level by 71.1%, 66.6% and 63.35 % respectively when compared to negative control		
					(iii) At doses of 25, 50 and 75 mg/kg of nerolidol increased superoxide dismutase activity by 31.1%, 34.8% and 66.1%, respectively when compared to negative control	(ii) Suggested to inactivate the enzyme nitric oxide synthase	[75]
					(iv) At doses of 25, 50 and 75 mg/kg of nerolidol increased catalase enzymatic activity by 109%, 148% and 177.7%, respectively when compared to negative control		
Antibacterial activity	Mixture of *cis*- and *trans*-nerolidol (Sigma Chemical Company, St. Louis, MO, USA)	-	*Staphylococcus aureus* FDA 209P, 14 strains of methicillin-susceptible *S. aureus* (MSSA) and 20 strains of methicillin-resistant *S. aureus* (MRSA)	Broth-dilution with shaking method (BDS)	Exhibited dose-related inhibition against 34 clinical isolates of *S. aureus.* Inhibitory dose 50% (ID_50_) ranged from 5.0 to 22.0 μg/mL and from 2.6 to 10.6 μg/mL against MSSA and MRSA respectively.	Suggested the aliphatic chain of nerolidol mediates the antibacterial activity by damaging the bacterial cell membrane	[76]
	Mixture of *cis*- and *trans*-nerolidol (Sigma Chemical Company, (St. Louis, MO, USA)	-	*Staphylococcus aureus* FDA209P	Broth dilution with shaking (BDS) method and quantitation of the leakage of K^+^ ions using K^+^-selective electrode	Treatment of nerolidol caused a dose-dependent increase in amount of K^+^ ions leakage from bacterial cells.	Mediates the antibacterial activity via cell membrane-distrupting mechanism and hence resulting in the leakage of K^+^ ions from bacterial cells	[77]
	Mixture of *cis*- and *trans*-nerolidol (Sigma Chemical Company, St. Louis, MO, USA)	-	*Staphylococcus aureus* FDA209P	Broth dilution with shaking (BDS) method and quantitation of the leakage of K^+^ ions using K^+^-selective electrode	(i) Caused a dose-dependent increase in K+ ions leakage from bacterial cells		[78]
					(ii) Exhibited minimum inhibitory concentration at 40 μg/mL		
	*trans*-Nerolidol	*Momordica charantia* L., seed	*Staphylococcus aureus* ATCC 6538	Broth microdilution method (MIC)	(i) Exhibited anti-microbial activity with MIC ranged from 125–500 μg/mL.	-	[7]
	Nerolidol (n.s.)	*Camellia sinensis* (L.) Kuntze, leaves	*Staphylococcus aureus* and *Streptococcus mutans*	Broth dilution method	Exhibited antibacterial activity against *S. aureus* and *S. mutans* with MIC measured at 200 and 25 μg/mL respectively	-	[79]
	Nerolidol (n.s.)	*Ginkgo biloba* L., leaves	*Salmonella enterica, Staphylococcus aureus* and *Aspergillus niger*	Disc-diffusion and broth dilution methods	(i) Exhibited antibacterial activity against *S. enterica*, *S. aureus* and *A. niger* with MIC, MBC and MFC values measured ranging from 3.9–15.6 μg/mL, 31.3–62.5 μg/mL and 62.5 μg/mL respectively.	-	[31]
	*cis*-Nerolidol and the racemic mixture of *cis*- and *trans*-nerolidol (Aldrich Chemical Co., Milwaukee, WI, USA)	-	*Escherichia coli* and *Staphylococcus aureus*	Agar-disc diffusion assay	Nerolidol (*cis*-nerolidol and the racemic mixture of *cis*- and *trans*-isomers) potentiated the action of antibiotics:	-	[80]
					(i) amoxicillin/clavulanic acid against *S. aureus* and		
					(ii) amoxicilline/clavulanic acid, ceftadizine and imipenem against *E. coli*		
	Nerolidol (n.s.) (Sigma, St. Louis, MO, USA)		*Escherichia coli* ATCC 25922 and *Staphylococcus aureus*	Disc-diffusion assay	(i) Nerolidol concentrations ranged from 0.5 to 2 mM enhanced the susceptibility of *S. aureus* to ciprofloxacin, clindamycin, erythromycin, gentamicin, tetracycline, and vancomycin	-	[81]
					(ii) Nerolidol (1 mM) enhanced the susceptibility of *E. coli* to polymyxin B		
	Racemic mixture of *cis*- and *trans*-nerolidol (1:1) (Aldrich, Madrid, Spain)	-	*Escherichia coli* ATCC 25922 and *Staphylococcus aureus* ATCC 25923	Antibiotic disc assay	Nerolidol (20 mM) potentiated the susceptibility of *E. coli* and *S. aureus* towards ciprofloxacin, erythromycin, gentamicin and vancomycin	-	[82]
Anti-biofilm activity	Mixture of *cis*- and *trans*-nerolidol	Black pepper, cananga, and myrrh EOs (Berjé (Bloomfield, NJ, USA), Jin Aromatics (Anyang, Gyeonggi Province, Korea) and Sigma-Aldrich (St. Louis, USA))	*Staphylococcus aureus*	Crystal violet biofilm assay	*Cis*-nerolidol at 0.01% (*v*/*v*) inhibited *S. aureus* biofilm formation by > 80 %; *trans*-nerolidol at similar concentration exerted 45% inhibition	-	[45]
	*trans*-Nerolidol	*Piper claussenianum* (Miq.) C. DC., leaves	*Candida albicans*	MTT assay	Concentrations of 0.06%–1.0% inhibited biofilm formation by 30% and 50% after 24 and 48 h incubation respectively	-	[32]
	*cis,trans-*Nerolidol and *cis-*nerolidol (Sigma Aldrich)	-	*Candida albicans*	MTT assay	1.0% of *cis,trans-*nerolidol exerted 76.1% reduction in the viability of pre-formed biofilm while only 67.0% reduction observed from 1.0% *cis*-nerolidol	-	[32]
Anti-fungal activity	Nerolidol (n.s.)	*Chamaecyparis obtusa* (Siebold & Zucc.) Endl. (Japanese cypress)	*Microsporum gypseum*	Broth microdilution method Skin lesion scoring in guinea pig model	(i) Exhibited MIC concentrations of 0.5%–2% against *M. gypseum*	-	[83]
					(ii) Nerolidol-treated group exhibited a significant improvement (*p <* 0.05) in lesion as compared to eugenol and econazole (positive control) treated groups		
	*trans*-Nerolidol	*Piper claussenianum* (Miq.) C. DC., Piperaceae, leaves	*Candida albicans*	Broth microdilution and trypan blue exclusion method	(i) Exhibited anti-fungal activity with MIC values ranging from 0.24% to 1.26%.	-	[32]
					(ii) Exhibited inhibitory effect on yeast-to-hyphae transition by 81%		
	Nerolidol (n.s.) (Sigma-Aldrich, Yongin, Korea)	-	*Trichophyton mentagrophytes*	Agar dilution method	Inhibited the hyphal growth of *T. mentagrophytes* at the concentration of 0.4 mg/mL.	-	[16]
	Nerolidol (n.s.)	*Camellia sinensis* (L.) Kuntze, leaves		Broth dilution method	Inhibited the growth of *T. mentagrophytes* at 12.5 μg/mL	-	[79]
	*trans*-Nerolidol	*Lantana radula* Sw., leaves	*Corynespora cassiicola*	Poison food (PF) technique	(i) *L. radula* EO at the concentration of 1000 mg/L and 3000 mg/L inhibited the growth of *C.* *cassiicola* by 17.2% and 40.6% respectively	-	[33]
					(ii) *L. radula* EO at the concentration of 5000 mg/L and 10,000 mg/L completely inhibited the growth of *C.* *cassiicola*		
	*trans*-Nerolidol	*Piper chaba* Hunter, leaves	*Fusarium oxysporum*, *Phytophthora capsici*, *Colletotrichum capsici*, *Fusarium solani* and *Rhizoctonia solani*	Spore germination assay and agar dilution method	Caused 55.1%–70.3% growth inhibition at concentration ranging from 125 to 500 µg/mL.	-	[34]
	*trans*-Nerolidol	*Warionia saharae* ex Benth. & Coss., aerial part	*Alternaria* sp., *Penicillium expansum* and *Rhizopus stolonifer*	Poisoned food (PF) technique and volatile activity (VA) assay	Inhibited the fungal spore production of *Alternaria* sp., *P. expansum* and *R. stolonifera* at 1, 2 and 2 µL/mL air respectively	-	[26]
	Nerolidol	*Allium sativum* L., bulb	*Sclerotium cepivorum*	Disc diffusion method; scanning electron microscopy	(i) Nerolidol ranged from 2.0 to 5.0 µg/disc displayed fungistatic property by inhibiting mycelial growth by ~85%	-	[84]
					(ii) Nerolidol ranged from 2.0 to 5.0 µg/disc inhibited the production of sclerotial by ~84%		
					(ii) Nerolidol at 4.0 µg/disc caused morphological alterations such as shorter branching, hyphal shrinkage and partial distortion		
Anti-trypanosomal activity	*trans*-Nerolidol	*Strychnos spinosa* Lam., leaves	*Trypanosoma brucei*	Alamar Blue™ assay.	Exhibited anti-trypanosomal activity with IC_50_ measured at 1.7 µg/mL (7.6 µM)	-	[35]
	*cis*-Nerolidol	*Leonotis ocymifolia* (Burm.f.) Iwarsson, aerial part		Trypanocidal and cytotoxic assays	Exhibited anti-trypanosomal activity with IC_50_ measured at 15.78 µg/mL	-	[27]
	Mixture of ±40% *cis*-nerolidol and ±55% of *trans*-nerolidol (Merck, Darmstadt, Germany)	-	*Trypanosoma evansi*	Collection of blood samples from *T. evansi*-infected mice for observation using light and electron microscopes	(i) Adverse morphological changes observed in nerolidol-treated group. The parasites lost their undulating membrane after 23 day post-treatment.	-	[85]
					(ii) Total disfigurement observed after 27 day post-treatment		
Anti-leishmanial activity	A mixture of *cis*- and *trans*-nerolidol	-	*Leishmania* (L.) *amazonensis*, *L. braziliensis*, and *L. chagasi*	MTT assay and metabolic labeling with [2-^14^C] mevalonic acid, [1-^14^C] acetic acid, [1(n)-^3^H] farnesyl pyrophosphate and l-[^35^S]methionine	(i) Inhibited the growth of *L. amazonensis, L. braziliensis* and *L. chagasi* promastigotes, and *L. amazonensis* amastigotes with IC_50_ of 85, 74, 75, and 67 µM respectively	Inhibition of the isoprenoid biosynthesis pathway	[86]
					(ii) Nerolidol at 100 µM reduced the percentage of intracellular parasitism of *L. amazonensis* by 95% from the pre-infected macrophages culture		
	*trans*-Nerolidol	*Baccharis dracunculifolia* DC., leaves	*Leishmania donovani*	Parasite lactate dehydrogenase (pLDH) assay, antileishmanial assay, schistosomicidal assay and cytotoxicity assay using the mammalian cells *Vero*.	Exhibited anti-leishmanial activity against promastigotes of *L. donovani* with IC_50_ and IC_90_ values of 42 and 85 µg/mL respectively.	-	[8]
	Nerolidol	*Piper claussenianum* (Miq.) C. DC., Piperaceae, leaves	*Leishmania amazonensis*	Protozoal arginase activity, nitrite determination and cytotoxicity assay using L929 fibroblast cells (mouse) and Raw cells (mouse macrophages)	(i) Nerolidol inhibited the arginase activity by 62.17% in the promastigotes of *Leishmania amazonensis*	Interferes with parasite-host cell interaction	[9]
					(ii) Nerolidol caused an increase in NO production (20.5%)		
	Nerolidol (n.s.) (Acros Organics, Geel, Belgium)	-	Promastigotes of *Leishmania amazonensis*	Anti-proliferative activity assay and electron paramagnetic resonance (EPR) spectroscopy of the spin-labeled 5-doxyl stearic acid	Nerolidol modulated the molecular dynamics of the lipid component in the *Leishmania* plasma membrane	Insertion of nerolidol into the lipid bilayer increased the fluidity of membranes, thus causing leakage of cytoplasmic content and eventually the death of *Leishmania* cells	[87]
Anti-schistosomal activity	Nerolidol (n.s.)	*Baccharis dracunculifolia* DC. (Asteraceae), leaves	*Schistosoma mansoni*	Schistosomicidal assay	100% mortality of *S. mansoni* adult worms after 24 h incubation with 10 to 100 mg/mL of EO containing nerolidol as the main constituent	-	[8]
	Racemic mixture of *cis*- and *trans*-nerolidol (1:1) (Sigma-Aldrich, St. Louis, MO, USA)	-		*In vitro* anti-schistosomal assay and microscopy studies	Exhibited anti-schistosomal activity by reducing worm motor activity and caused 100% mortality of male and female schistosomes at concentration of 31.2 and 62.5 µM respectively	(i) Induced severe tegumental damage in adult schistosomes.	[88]
						(ii) Caused alterations on the tubercles of male parasites	
Anti-malarial activity	Nerolidol (n.s.)	*Virola surinamensis* (Rol. ex Rottb.) Warb., leaves	*Plasmodium falciparum*	*In vitro* anti-plasmodial assay	Treatment with 100 µg/mL of nerolidol caused 100% inhibition in the development of young trophozoite to the schizont stage after 48 h	-	[29]
	*trans-*Nerolidol	*Piper claussenianum* (Miq.) C. DC., leaves			Exerted anti-malarial activity with IC_50_ of 11.1 μg/mL	-	[89]
	Nerolidol (n.s.) (Sigma, St. Louis, MO, USA)	-		Immunoprecipitation assays and metabolic labeling	Exhibited inhibitory activity on the biosynthesis of the isoprenic side chain of the benzoquinone ring in ubiquinones during the schizont stage	Interferes with the elongation of isoprenic chains via inhibition of isoprenyl diphosphate synthases	[90]
	Nerolidol (n.s.) (Sigma, St. Louis, MO, USA)	-			Nerolidol at 50 nM inhibited the synthesis of the isoprenic chain attached to coenzyme Q at all intraerythrocytic stages	-	[91]
	Nerolidol (n.s.)	-		Isobolographic analysis	Nerolidol mediated supra-additive (the sum of the fractions of IC_50_ of < 1) interaction with fosmidomycin and squalestatin with average IC_50_ values of 0.57 and 0.62 µM, respectively in the inhibition of plasmodial isoprenoid pathway	-	[92]
Other anti-parasite activities	Mixture of *cis*- and *trans*-nerolidol (Sigma-Aldrich, St. Louis, MO, USA)	-	Four *Babesia* species (*B. bovis, B. bigemina, B. ovata,* and *B. caballi*)	*In vitro* growth inhibition assay	Inhibited *in vitro* growth of *B. bovis*, *B. bigemina*, *B. ovata*, and *B. caballi* with IC_50_ values of 21 ± 1, 29.6 ± 3, 26.9 ± 2, and 23.1 ± 1 µM respectively	Inhibits the isoprenoid biosynthesis pathway in a similar mechanism with that of *P. falciparum*	[93]
	Mixture of *cis*- and *trans*-nerolidol (Sigma Chemical Company, St. Louis, MO, USA)	-	*Caenorhabditis elegans*	Mortality assay against *Caenorhabditis elegans*	Caused 74.0% mortality of *C. elegans* at 50 µg/mL	-	[94]
	Nerolidol (n.s.)	-	L_3_ larvae of *Anisakis*	*In vitro* and *in vivo* larvicidal activity	(i) Nerolidol at both 31.5 and 62.5 µg/mL resulted in 100% mortality of L_3_ larvae of *Anisakis* type I after 4 h.	-	[95]
					(ii) Only 20% of nerolidol-treated rats were affected by gastric wall lesions caused by *Anisakis* larvae in comparison to 86% of the control rats		
Insecticidal activity	*trans*-Nerolidol	Siam-wood (*Fokienia hodginsii* (Dunn) A.Henry & H H.Thomas), wood	Mosquito and house flies	House fly toxicity test	Exhibited insecticidal activity with LD_50_ measured at 0.17 µmol/fly	-	[51]
	Combination of nerolidol (n.s.) and linalool	*Capparis tomentosa*, leaves	Maize weevil (*Sitophilus zeamais*)	Repellency assay using a glass Y-tube Olfactometer	Exhibited mean repellency value of 58.23% ± 2.95% against *S. zeamais* at 2 µL	-	[30]
	Nerolidol (n.s.) (Moellhausen SpA,Vimercate, Milano, Italy)	*Melaleuca alternifolia* (Maiden & Betche) Cheel (tea tree oil)	*Pediculus capitis* (head lice) and its eggs	Pediculicidal and ovicidal activities	Nerolidol in combination with tea tree oil with ratio of 1:2 (tea tree oil 0.5% plus nerolidol 1%), exerted a total killing effect of lice within 30 min and abortive effect of louse eggs after 5 days.	-	[96]
	Nerolidol (n.s.)	*Magnolia denudata* Desr., seeds	*Culex pipiens* pallens, *Aedes aegypti*, *Aedes albopictus and Anopheles sinensis*	Direct-contact mortality bioassay	Exerted larvacidal activity against *Culex pipiens pallens*, *Aedes aegypti*, *Aedes albopictus* and *Anopheles sinensis* with LD_50_ value of 9.84, 13.85, 16.34 and 20.84 mg/L respectively	-	[47]
	*trans*-Nerolidol	*Melaleuca quinquenervia* (Cav.) S.T.Blake, leaves	*Aedes aegypti*	Larvicidal activity test	Exerted larvicidal activity with ≥ 95% and > 80% mortality of *A. aegypti* at 0.1 mg/mL and 0.05mg/mL respectively	-	[36]
		*Piper aduncum* L., leaves	*Tetranychus urticae* Koch	Fumigant, contact, repellency and two-choice assay	Exerted acaricidal activity with repellency value of 83.2% ± 0.59 % at 9.8 µg/mL	-	[37]
	Nerolidol (n.s.)	*Baccharis dracunculifolia* DC., leaves	*Rhipicephalus microplus*	Larval packet test (LPT) and engorged female immersion test	(i) Exerted acaricidal activity when concentration more than 5mg/mL and 100% mortality of larvae at 15 mg/mL	-	[97]
					(ii) Reduced the quality of the egg and larval hatching rate with increasing concentration from 20 to 50 mg/mL		
Antiulcer activity	Nerolidol (n.s.)	*Baccharis dracunculifolia* DC., leaves	-	*In vivo* antiulcer activity in male Wistar rat ulcer models induced with ethanol, indomethacin and stress	Nerolidol displayed gastroprotective activity by inhibiting the formation of ulcers induced by all physical and chemical agents in dose-dependent manner (50, 250, 500 mg/kg)	-	[98]
Skin penetration enhancer activity	Nerolidol (n.s.) (Aldrich, Gillingham, UK)	-	-	*In vitro* diffusion studies and stratum corneum-water partitioning studies	Increased diffusion rate by over 20-fold for transdermal delivery of drugs such as 5-fluorouracil	Nerolidol exhibits a chemical structure that allows it to align within the lipid lamellae of the stratum corneum in order to disrupt the organization of stratum corneum	[99]
	Nerolidol (n.s.) (Alfa Aesar Ltd., Haverhill, MA, USA)	-	-	Solubility studies, *ex vivo* permeation studies and histopathological studies	The enhancement effect is increased with the increasing lipophilicity; the rank of order (nerolidol > farnesol > limonene > linalool > geraniol > carvone > fenchone > menthol) in facilitating transdermal delivery of alfuzosin hydrochloride		[100]
	Nerolidol (n.s.) (Merck-Schuchardt, Hohenbrunn, Germany)	-	-	*In vitro* permeation studies	Exhibited the highest permeation enhancing ability with a 3.2-fold increase in permeation of selegiline hydrochloride across the rat skin, followed by the effect of carvone (2.8-fold increase) and anethole (2.6-fold increase)	-	[101]
	Nerolidol (n.s.) (Aldrich Chemical Co. Milwaukee, WI, USA)	-	-	*In vitro* skin permeability studies	Most effective terpene enhancer for percutaneous permeation of four different drug models (nicardipine hydrochloride, hydrocortisone, carbamazepine, and tamoxifen) when compared to fenchone, thymol and limonene	-	[102]
Anti-nociceptive and anti-inflammatory activities	*trans*-Nerolidol	*Peperomia serpens* (Sw.) Loudon, leaves	-	(i) Chemical (acetic acid and formalin) and thermal (hot plate) models of nociception	*trans-*Nerolidol could be responsible for the anti-inflammatory and anti-nociceptive effects displayed by essential oils of both *Peperomia serpens* (Sw.) Loudon and *Piper aleyreanum* C. DC	-	[38]
				(ii) Carrageenan- and dextran-induced paw edema tests in rats croton oil-induced ear edema			
				(iii) Cell migration, rolling and adhesion activities			
	*trans*-Nerolidol	*Piper aleyreanum* C. DC, aerial parts	-	(i) Nociception induced by formalin		-	[10]
				(ii) Evaluation of locomotor activity			
				(iii) Induction of acute gastric lesions			
	Nerolidol (n.s.) (Sigma, St. Louis, MO, USA)	-	-	(i) Rotarod, acetic acid-induced writhing, formalin and hot-plate tests (ii) Involvement of ATP-sensitive opioid and GABAergic K^+^ channels (iii)Carrageenan-induced paw edema (iv) Analysis of leukocytes, tumor necrosis factor (TNF-α), interleukin 1 beta (IL-1β) and interleukin 6 in peritoneal lavage	(i) For acetic acid-induced writhing test, at the doses of 200, 300 and 400 mg/kg, nerolidol reduced the frequency of acetic acid-induced writhing at all three doses tested compared to the mice in the control group (55% ± 1.1%, 53% ± 4.5%, and 41% ± 2.4%, respectively) (ii) For formalin test, at the doses of 200, 300 and 400 mg/kg, nerolidol significantly inhibited licking time by 20% ± 3.3%, 33% ± 5.9% and 37% ± 4.8%, respectively when compared to the control mice. (iii) For hot-plate test, no increase in the reaction time to painful stimulation in the mice treated with nerolidol when compared to the control mice. (iv) Reduced leukocytes level by 51% ± 0.7%, 37% ± 0.5% and 57% ± 0.4% at doses of 200, 300 and 400 mg/kg respectively (v) Reduced the level of tumor necrosis factor (TNF-α) at doses of 300 (59.3% ± 30.2%) and 400 (62.2% ± 13.7%) in peritoneal lavage. (vi) IL-1β production was inhibited after treatment with nerolidol (1, 10, 50 and 100 µM) whereas IL-6 level was unchanged	(i) Anti-nociceptive activtity of nerolidol was indicated to be mediated by GABA_A_ receptors, as the use of bicuculline, a GABA_A_ antagonist inhibited the effect of nerolidol in reducing the paw licking times (ii) Anti-inflammatory activity of nerolidol was suggested to be mediated by inhibiting the production or the activity of pro-inflammatory cytokines such as TNF-α analgesic and IL-1β	[103]
Anti-cancer or anti-tumor activity	Nerolidol (a combination of *cis*-nerolidol 40.7%, *trans*-nerolidol 58.3%, *cis*-dihydronerolidol 0.4% and *trans*-dihydro-nerolidol) (Kurt Kitzing Co. Wallerstein, Germany)	-	-	Cytotoxicity assay on HeLa cell lines using CytoTox-96^®^-assay	Exhibited anticancer effect against HeLa cells with CC_50_ value at 1.5 ± 0.7 µM	-	[104]
	*cis*-Nerolidol (Charabot S.A. Grasse, France)	-	-	Cytotoxicity and cytoproliferative activity on HeLa cell lines using Cytotoxicity Detection Kit (LDH) and the Cell Proliferation Reagent WST-1, respectively	Exhibited cytotoxic effect (16.5 ± 6.7 μM) against HeLa cells	-	[105]
	Nerolidol (n.s.)	*Camellia sinensis* (L.) Kuntze, leaves	-	MTT assay	Exhibited cytotoxic effect with IC_50_ value of 2.96 and 3.02 µg/mL against BT-20 breast carcinoma and HeLa cells respectively	-	[106]
	*trans-*Nerolidol	*Zornia brasiliensis* Vogel, leaves	-	*In vitro* cytotoxic activity assay using Alamar blue assay, and *in vivo* antitumor activity assay	(i) *trans*-Nerolidol induced cytotoxic effect on B16-F10, HepG2, HL-60 and K562 cells with IC_50_ value of >25, >25, 21.99 and 17.58 µg/mL respectively	-	[39]
					(ii) The EO at dose of 100 mg/kg containing *trans*-nerolidol as major constituent reduced the weight of tumor in mice injected with B16-F10 melanoma by 38.61%		
		*Myrica rubra* (Lour.) Siebold & Zucc.*,* leaves	-	Neutral red uptake (NRU) test, MTT assay and 2′,7′-dichlorodihydrofluorescein-diacetate (H2DCF-DA) oxidation	Potentiated the action of doxorubicin, an anticancer drug in the modulation of CaCo-2 cancer cells	-	[40]
	Nerolidol (n.s.) (Sigma Aldrich Chemical Company)	-	-	*In vivo* anti-cancer study	(i) Reduction of incidence of intestinal neoplasia from 82% to 33% in rats fed with nerolidol	Modulation of nerolidol on protein prenylation which responsible for the formation of cancer	[107]
					(ii) Reduction of number of tumors/rat from 1.5 to 0.7 in rats fed with nerolidol		
	Combination of farnesol and nerolidol (n.s.)	-	-	*In vitro* anti-cancer study	The combination suppressed the proliferation of human HL-60 acute promyelocytic leukemia (HL-60) cells by 20%. Meanwhile, farnesol isomers (2.5 µmol/L) and nerolidol (5 µmol/L) individually suppressed the proliferation of HL-60 cells by 4 and 9%, respectively	Nerolidol induced cell cycle arrest at the G_0_-G_1_/S interphase in HL-60 cells and eventually lead to apoptotic cell death	[108]
	*trans-*Nerolidol	*Myrica rubra* (Lour.) Siebold & Zucc.*,* leaves	-	Cell adhesion and apoptosis luminescent assays	(i) Reduced adhesion of HT29 to collagen.	Nerolidol induced apoptosis in cancer cells	[109]
					(ii) Suppressed cell adhesion of HT29 cells in the presence TNFα cytokines		
					(iii) Decreasing the phosphorylation of NF-κB and increased the activity of caspases		

Key: n.s. = not specified**.**

**Table 5:** Comparison of pharmacokinetic studies of nerolidol conducted by Saito *et al.* and He *et al.* [22,25]

Parameters	Saito *et al.* [22]	He *et al.* [25]
Type of nerolidol	Mixture of *cis*- and *trans*-nerolidol (1:3)	Mixture of *cis*- and *trans*-nerolidol (2:3)
Analytical method used	GC-MS	LC-MS
Animal used	BALB/c mice	Sprague-Dawley rats
Route of administration	Oral	Intraperitoneal injection
Dosage (mg/kg)	1000	25
Type of sample used	Plasma	
Time collection taken (min)	30, 60, 120, 180, 240, 300, 360, 480 and 720	10, 20, 30, 60, 90, 120, 240 and 360
Peak plasma concentration (C_max_) (µg/mL)	~0.27 ± 0.07	8.30 ± 1.07
Peak time (T_max_) (min)	30	20
Elimination half life (T_1/2_) (min)	n.a.	20.98 ± 7.71
Mean residence time (MRT) (min)	n.a.	27.72 ± 2.14
Clearance (L/min/kg)	n.a.	0.082 ± 0.012
Time for drug to be eliminated to almost near zero	12	~2
Human equivalent dose ^a^ (HED) (mg/kg)	81.08	4.05

Key: (i) n.a. = data not available; (ii) a = HED values were calculated based on the formula for dose translation based on body surface area (BSA) [156] as follows: Human equivalent dose (HED) (mg/kg) = Animal dose (mg/kg) × $\frac{Animal\;Km}{Human\;Km};\;$with adult human, rat and mouse K_m_ factors of 37, 6 and 3 respectively [156].
